# Masking, crowding, and grouping: Connecting low and mid-level vision

**DOI:** 10.1167/jov.22.2.7

**Published:** 2022-02-11

**Authors:** Josephine Reuther, Ramakrishna Chakravarthi, Jasna Martinovic

**Affiliations:** 1 School of Psychology, University of Aberdeen, UK; 2 Department of Psychology, School of Philosophy, Psychology and Language Sciences, University of Edinburgh, UK

**Keywords:** masking, crowding, grouping, receptive fields, integration fields, association fields, mid-level fision

## Abstract

An important task for vision science is to build a unitary framework of low- and mid-level vision. As a step on this way, our study examined differences and commonalities between masking, crowding and grouping—three processes that occur through spatial interactions between neighbouring elements. We measured contrast thresholds as functions of inter-element spacing and eccentricity for Gabor detection, discrimination and contour integration, using a common stimulus grid consisting of nine Gabor elements. From these thresholds, we derived a) the baseline contrast necessary to perform each task and b) the spatial extent over which task performance was stable. This spatial window can be taken as an indicator of field size, where elements that fall within a putative field are readily combined. We found that contrast thresholds were universally modulated by inter-element distance, with a shallower and inverted effect for grouping compared with masking and crowding. Baseline contrasts for detecting stimuli and discriminating their properties were positively linked across the tested retinal locations (parafovea and near periphery), whereas those for integrating elements and discriminating their properties were negatively linked. Meanwhile, masking and crowding spatial windows remained uncorrelated across eccentricity, although they were correlated across participants. This suggests that the computation performed by each type of visual field operates over different distances that co-varies across observers, but not across retinal locations. Contrast-processing units may thus lie at the core of the shared idiosyncrasies across tasks reported in many previous studies, despite the fundamental differences in the extent of their spatial windows.

## Introduction

Visual processing is not local. The processing and perception of input from one location is affected by the presence and processing of input at a wide range of other locations (Albright & Stoner, 2002; Spillmann et al., 2015; Zipser et al., 1996). Suppression from adjacent stimuli can stem from masking, a low-level contrast-driven process thought to occur within perceptive fields (Spillmann, 2014). Suppression can also stem from crowding, a higher level, featural process that interferes with discrimination and recognition. Crowding is thought to occur within integration fields (Pelli et al., 2004). In contrast, there are facilitatory contextual influences that enable discrete featural elements to be grouped into larger wholes. Association fields explain such integration of oriented lines into contours (Field et al., 1993). Thus, at least three different constructs have been invoked to account for interactions of visual processing across space: I) perceptive fields, II) integration fields, and III) association fields, each of which reflects a different aspect of spatial vision. These fields explain key low and mid-level vision processes by referring to the underlying neural substrate—putatively, a field—in a nod to the well-known neural concept of a receptive field. Visual fields determine the extent of information that is processed within different neural units in the visual cortex. Thus, they determine which information will be bound together and which will be individuated.

Perceptive fields are psychophysical equivalents of receptive fields and play a crucial role in low-level, contrast-based processing (Jung & Spillmann, 1970; Spillmann, 2014). They are presumed to be situated in the primary visual cortex and deal with the basic extraction of features, for example, orientation or spatial frequency (Anderson & Burr, 1987; Polat & Sagi, 1993; Tolhurst & Barfield, 1978). Integration fields likely pool over the features that were processed in perceptive fields (Harrison & Bex, 2015; Levi et al., 2002; Pelli et al., 2004; Van den Berg et al., 2010). They are assumed to be the locus of a process known as visual crowding (Pelli et al., 2004), which leads to a reduction in the ability to discriminate attributes of objects (e.g., their orientation) when there are other objects nearby (Levi, 2008). Thus, they determine the spatial windows in which elements can be integrated into a common texture, thereby losing individual features (Parkes et al., 2001). There is considerable debate about where these computations might be implemented, but a reasonable venture would be that it is likely to involve multiple sites in the visual cortex (V1–V4; Anderson et al., 2012; Chen et al., 2018; Freeman et al., 2011; Millin et al., 2014). Association fields, like integration fields, constrain advanced processes crucial for the perceptual organisation of visual scenes by linking individual features into contours (Field et al., 1993) and thus forming them into an object—that is, a figure, rather than a texture or a background. Association fields also pool over the output of feature detectors and are assumed to be implemented in V2 to V4 (Hess et al., 2014; Mijović et al., 2014; Shpaner et al., 2013), yet seem to have a larger spatial range than integration fields.

Perceptive, integration, and association fields have one common aspect: the processing of potentially large-scale information within fields of flexible size. Perceptive field sizes have been estimated using a range of methods, leading to comparable estimates. Studies using the suppressive zone of lateral masking tasks as an estimate of perceptive field (Lev & Polat, 2011; Polat & Sagi, 1993) yield results in line with estimates derived from prior psychophysical work (e.g., Ransom-Hogg & Spillmann, 1980; Watson et al., 1983; Westheimer, 1967), human and animal physiology (Hubel & Wiesel, 1960; Oehler, 1985; Spillmann et al., 1987), and reverse correlation techniques (Neri & Levi, 2006). Perceptive field size is between 18’ of arc (i.e., $∼0.3{}^{\circ }$) and 1${}^{\circ }$ in the fovea (Lev & Polat, 2011; Ransom-Hogg & Spillmann, 1980; Spillmann, 1971) and increases with eccentricity (Harvey & Dumoulin, 2011; Lev & Polat, 2011; Spillmann, 1994; Yazdanbakhsh & Gori, 2008). It is modulated by spatial frequency (Anderson & Burr, 1987), intensity of the adapted light level (Troscianko, 1982; Troup et al., 2005), and size (Pelli et al., 2004). Conversely, integration field size does not scale with spatial frequency (Chung et al., 2001; Levi et al., 2002), or size (Tripathy & Cavanagh, 2002), yet scales with eccentricity (Toet & Levi, 1992). The spatial extent of visual crowding, which is commonly used to estimate integration field size, is routinely quoted to be one-half the target's eccentricity (Bouma, 1970; Pelli & Tillman, 2008). However, it can range from as little as 0.13 to 0.80 times the target eccentricity (e.g., Chung et al., 2001; Soo et al., 2018; Strasburger & Malania, 2013; also see Pelli et al., 2004). Contour integration or grouping within the putative association field is modulated by eccentricity (Nugent et al., 2003), orientation (Dakin & Baruch, 2009; Field et al., 1993), and spatial frequency (Beaudot & Mullen, 2003; Dakin & Hess, 1998), and is therefore dependent on some of the same information as masking and crowding. Critical separation as a measure of distance over which adjacent elements are combined into a contour ranges from around one degree at high spatial frequencies to several degrees at low spatial frequencies (Beaudot & Mullen, 2003; Dakin & Hess, 1998).

Superficially, it may appear that masking, crowding, and contour integration are related because they all depend on eccentricity. But, this logic is unsatisfactory (because most visual processes vary by eccentricity) and may be misleading. For example, masking and contour integration both depend on element orientation and spacing. This may be taken to suggest a connection between the processes. However, comparing the influence of monocular, binocular, dichoptic, and stereoscopic presentation on flanker facilitation and contour integration, the former was attributed to earlier sites (V1), while the latter is thought to occur at subsequent processing stages (V2); (Huang et al., 2006). Certain models suggest that contour integration is the result of recurrent processing, especially in situations that allow multiple viable ways to combine elements into contours or objects (Houtkamp & Roelfsema, 2010). Such incremental grouping is modulated by Gestalt cues like connectedness and collinearity (Kapadia et al., 1995; Roelfsema, 2006; Roelfsema et al., 1998). This may also explain the ability of grouping to modulate crowding, resulting in strong crowding when target and flankers are grouped together and weak crowding when flankers group separately from the target (Livne & Sagi, 2007; Manassi et al., 2013; Wolford & Chambers, 1983). Although masking, crowding, and grouping may or may not have a common source, it is possible that downstream fields (integration and association fields) inherit the outputs and hence some characteristics of upstream fields (perceptive fields). In contrast, each type of field might introduce its own idiosyncratic characteristic, which might render their representations and processing distinct and uncorrelated with their respective upstream versions.

Several research programs have investigated potential links between masking, crowding, and grouping (e.g., Chakravarthi & Pelli, 2011; Dakin & Baruch, 2009; Doron et al., 2015; Lev & Polat, 2015; May & Hess, 2007). Lev and Polat (2015) characterized the relations between masking and crowding. Up to that point, these processes had usually been studied separately (Pelli et al., 2004; Petrov et al., 2007, 2005) because they were considered distinct phenomena. It is important to note that, even when they were examined and compared in the same study, the stimuli used for each task were typically dramatically different (e.g., Petrov et al., 2007). Lev and Polat (2015) assessed perceptive field size, and the magnitude of crowding and masking, and found that the deleterious effects of crowding were correlated with perceptive field size, particularly when the target-flanker spacing was the same as the estimated perceptive field size. In the periphery, crowding was stronger for participants with larger perceptive field sizes for both simultaneous (spatial crowding) and asynchronous (temporal crowding) stimulus presentation. A quantitative framework based on neural inhibition accounts for these findings and demonstrates that there is a common neural substrate for processes within perceptive and integration fields. In contrast, Chakravarthi and Pelli (2011) linked crowding and grouping, finding that they might be two sides of the same coin (also see work from the Herzog lab, e.g., Francis et al., 2017; Herzog et al., 2015). Their study manipulated good continuation between Gabor stimuli and discovered that the pattern of results for the contour integration task mirrored that in the crowding task. When performance in one task improved, performance in the other deteriorated. They, therefore, argued that the binding between elements induced by both contour integration and crowding was the same, except that it was beneficial in the contour integration task, whereas it was detrimental in the crowding task. This finding suggests that processes within integration and association fields may be tightly linked.

Although masking has been linked to crowding, and crowding has been linked to contour integration, the specific interactions among all three processes are still largely unmapped, leaving models of mid- and high-level vision lacking in that aspect. Therefore, the aim of this study is to build a common framework that will encompass neural processing within these three types of visual fields. To do so, we have developed a nine-element Gabor grid that enables us to test masking, crowding, and grouping without major changes in stimulus configuration, simply by instructing participants to perform a different task (target detection, discrimination, and integration) while modulating inter-Gabor spacing and eccentricity. The advantage of this simple, nine-element stimulus is that it maintains the spread of spatial attention within an area of space that is constant across tasks. There are large differences in task requirements across masking, crowding, and grouping. Specifically, contour integration requires attention to be spread across all elements that form a contour; in contrast, crowding and masking require attention to be focused on one element, while ignoring the rest. To minimize this confound, we specified a configuration of elements that keeps the spread of attention roughly constant across tasks, remaining within the spatial area defined by only nine elements. The detection, discrimination, and integration tasks that can be performed with this stimulus may be a slight departure from those traditionally used to study masking, crowding, or grouping, but still target the appropriate mechanism. We use contrast as the dependent measure, which allows for the determination of the minimal (baseline) contrast necessary to perform the respective tasks as well as the spatial extent over which task performance is stable. This spatial window can be taken as an indicator of field size, where elements that fall within a putative field are readily combined. For tasks that rely on element individuation (masking and crowding), threshold elevation above baseline contrast indicates that non-target elements are starting to encroach on the target, while for tasks that rely on integration (grouping) threshold elevation indicates that elements resist being readily combined.

Given what is already known from the summarized literature, we expect the spatial windows to become progressively larger along the processing stream, with perceptive fields having the smallest extent, integration fields having a moderate extent, and association fields having either the same size as integration fields or being larger. Of interest would be the qualitative and quantitative relationships among the characteristics of the three fields. One possibility is that processing stages inherit characteristics from earlier steps in the processing stream because they rely on the same retinotopic input. For example, Greenwood et al. (2017) assessed crowding, spatial localization, and saccadic precision in the same set of participants and observed a pattern of between-subject correlations, with lower level spatial vision acting as a predictor for both crowding and saccades. Surprisingly, despite these correlations, these processes showed clear dissociations, indicative of independent spatial representations. The authors suggested that idiosyncratic variations in spatial topology (i.e., retinotopic cell density or receptive field size) are inherited by downstream areas. However, this inheritance can occur even when the processes in question do not share a common spatial representation. All these possibilities predict that the range of interactions in the three fields would be strongly correlated. Alternatively, the characteristics might be distinct for each of the fields as they implement different kinds of processing. These differences would reflect differences in the computations undertaken in these fields. We will evaluate these key predictions by 1) characterizing distinctive attributes of masking, crowding and grouping using linear mixed effect (LME) models fitted separately to data from detection, discrimination and integration tasks and 2) examining correlations between contrast and spatial window constraints of each process, measured from a relatively large (*n* = 40) participant sample. In addition to commonly used between-participant correlations, which interrogate variability across participants on data that is either averaged or aggregated across eccentricities (c.f. Greenwood et al., 2017), we intend to evaluate within-participant correlations across eccentricity (Bakdash & Marusich, 2017). Masking, crowding, and grouping are all affected by eccentricity. Therefore, within-participant patterns of variability that emerge consistently across the three eccentricities (3.5, 7, and 10.5 degrees) could be taken as robust evidence in favour of processing commonalities that operate across the parafovea and the near periphery.

## Methods

### Overview

Over the course of three to four sessions, each lasting 1.5-2.0 hours, we took several baseline measurements for central and peripheral vision, and assessed contrast thresholds for a stimulus configuration of oriented Gabors under the influence of masking, crowding and grouping. In the first testing session we assessed the following visual functions: 1) visual acuity at the testing distance, 2) far visual acuity, 3) contrast sensitivity function at the testing distance, and contrast detection thresholds in the periphery using 4) an adjustment procedure and 5) a threshold procedure. These baseline measures were followed by the first main task: assessing contrast thresholds for grouping. Subsequent to completing the grouping task, participants were presented with 6) the masking task and 7) the crowding task in sessions two to four. The final session ended with 8) an adjustment task determining suprathreshold perceived contrast matches at the different eccentricities used in the experiment. The number of sessions and their duration was adapted in accordance with participants’ preferences to avoid fatigue. Regular brief breaks were taken by the participants for the same purpose. Finally, participants over 60 years of age also completed the Montreal Cognitive Assessment (MoCA).

### Participants

Forty participants with a mean age of 42.6 years (SD 14.3) completed all parts of the current study. Five additional participants started the study, but did not complete all parts. Participants were recruited from the general population by word of mouth, from the staff and student population of the University of Aberdeen via internal online notice boards and mailing lists, as well as from a participant panel via email. All participants had normal or corrected to normal vision for the testing distance of 57 cm. To correct for ametropia (e.g., short-sightedness, astigmatism, and reduced near-accommodation) participants wore their own contact lenses or glasses. In cases where participants had no glasses that were specifically suited for the testing distance, a trial frame and test lenses were used. This was done for those participants who only had varifocals and bifocals—these types of glasses were not deemed adequate due to their restriction of near vision to the central visual field when eye and head movements are not permitted (Charman, 2014), and for participants who did not own single distance glasses that were adequate for the testing distance (*n* = 4). Eight participants completed the Montreal Cognitive Assessment. All achieved scores in the normal range (≥26; mean, 28.75 ± 0.89). Participants gave informed written consent to take part and were reimbursed for their time and effort. The study was approved by the ethics board for the School of Psychology at the University of Aberdeen and was in line with the Declaration of Helsinki.

### Apparatus

A 32” Display++ LCD monitor (CRS, Milton Keynes, UK) with a resolution of 1920×1080 pixels, a pixel size of 0.37 mm, a refresh rate of 120 Hz, and maximal luminance output of 118.1 cd/m^2^ was used as the primary screen for stimulus presentation. Temporal dithering algorithms were applied to achieve 16-bit gray values in the mono++ setting. The screen was controlled by a Dell Precision T1700 computer with a Nvidia Quadro K420 graphics card. Stimulus presentation was implemented in Matlab (Mathworks, Natick, MA) using Psychtoolbox functions (Brainard, 1997; Kleiner et al., 2007; Pelli, 1997) and the Palamedes toolbox (Prins, 2018). For the assessment of contrast sensitivity functions only, a 21” ViewSonic Professional Series P227f CRT monitor with a resolution of 1024×768 pixels and a refresh rate of 100 Hz was used. This screen was controlled using the ViSaGe MKII Stimulus Generator (CRS) and a Dell Precision T3500 computer, running the Visual Psychophysics Engine (CRS). Where stated, central fixation was monitored using a magnifying camera system and a transparent screen integrated with the chin and forehead rest. The camera provided a live stream of the reflection of the observer's eye to an AD 910A Video Monitor (Sensormatic Electronics Corporation, Boca Raton, FL) with a refresh rate of 50 Hz, which was controlled by a Model 5000 Control Unit (ASL). A LiveTrack Fixation Monitor (CRS) was used as an infrared light source. Fixation was monitored manually by the experimenter. Trials were rejected online by key release (standard keyboard). Responses were primarily recorded via a 5-key button box (Cedrus RB-530, Cedrus Corporation, Los Angeles, CA), which was placed under a black monitor stand with the infrared light source resting on top of it. For the assessment of the contrast sensitivity function and far visual acuity standard keyboards were used. An Early Treatment Diabetic Retinopathy Study (ETDRS) intermediate distance chart for a distance of 66 cm was used to assess visual acuity (VA). At the adjusted testing distance (57 cm), it allows visual acuity to be assessed between 1.36 and −0.24 logMar, which approximates to 6/140 and 6/3.5, respectively. The chart was evenly illuminated and its white background reflected 93.5 cd/m^2^. The letter test of the Freiburg Vision Test (FrACT; Bach, 1996, 2006) was used to assess far visual acuity.

### Stimuli and procedure: Control measurements

#### Visual acuity (near)

To assess near visual acuity, participants were seated 57 cm from the ETDRS-chart (SLOAN optotypes) and were instructed to read the first letter of each line starting from the top. A reduction in reading speed was considered to indicate an increase in the difficulty to identify the letters. Once this point was reached, participants were asked to read full lines of letters, starting with the line two lines up from where they were stopped. This was indicated by naming the first letter of this line (e.g., “Please read all letters of the line that starts with an O.”). Participants were then asked to read each of the following lines until they reported fewer than three letters of a line correctly. This approach was used to keep test time short while still ensuring that participants read at least one complete line correctly. Visual acuity was calculated from individual letter accuracy.

#### Visual acuity (far)

To assess far visual acuity the letters of the FrACT (Bach, 1996, 2006) were presented on the Display++ at a viewing distance of 3 m. This test was used for consistency since it uses the same letters as the ETDRS chart. At the testing distance of 3 m visual acuity can be assessed up to 2.13 (MAR^−1^; approximately −0.32 logMAR) on the Display++. Participants were presented with one letter at a time, with 24 trials in total. FrACT uses a best PEST procedure assuming a constant slope on a logarithmic acuity scale to determine the size of the next presented letter based on the last response. However, every sixth letter was presented at a size four times the current threshold to reduce frustration and to end the trial run on a positive. Participants responded verbally and the experimenter keyed in their response using a standard keyboard. If participants reported to be unsure, they were encouraged to report their best guess.

#### Contrast sensitivity function (CSF)

The contrast sensitivity function was assessed using seven spatial frequencies (SF: 0.3, 0.5, 1, 2, 4, 6, and 8 cycles per degree or cpd) in an adjustment task. Participants were seated at a viewing distance of 57 cm from the screen. Vertically oriented sine-wave stimuli within a circular aperture of 10${}^{\circ }$ visual angle were presented in the center of the screen on a background metameric with illuminant C and set to 20 cd/m^2^. Stimuli reversed in polarity at a rate of 0.5 Hz. While fixating the grating, participants were instructed to adjust its contrast to the point where the grating itself and/or the change of contrast polarity of the grating were just noticeable. Participants used the up and down arrow of a standard keyboard to make the adjustments and confirmed their response by pressing the spacebar, which concluded a trial. A beep indicated that a new grating was presented. The order of spatial frequencies was low to high. The sequence of seven trials (one per SF) was presented thrice leading to 21 trials in total.

#### Stimuli and procedure: Main experiments

**Figure 1.**
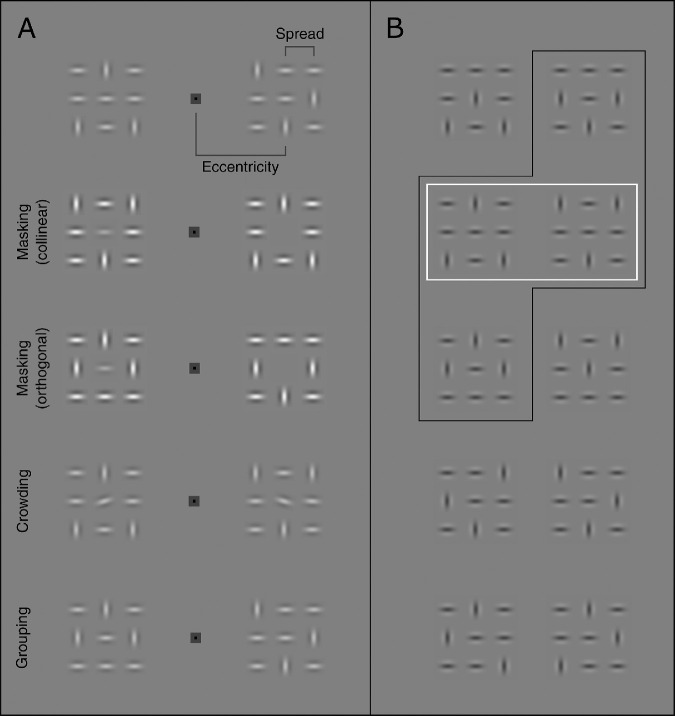
Grid-stimuli used in the main experiments. Each grid-stimulus consisted of nine Gabor patches - three were oriented vertically and six horizontally. Vertically oriented Gabors were spread across the 3 × 3 grid in such a way that they were never horizontally or vertically adjacent to one another, and were never positioned along the main diagonals. (A) Examples of the stimuli as they were presented on a given trial for each of the tasks, respectively. As shown in the examples, simultaneously presented stimuli always had the same contrast polarity. All trial examples are shown with the target stimulus to the left and the distractor stimulus to the right of the fixation mark. Target stimuli were defined by the presence of the central element for the masking task (target: central element present, distractor: central element absent), by the tilt of the central element for the crowding task (target: left tilted, distractor: right tilted), and by horizontal alignment of the horizontal Gabor elements (target: 3 elements, distractor: 2 elements of a row horizontally aligned). Note that each distractor grid in the grouping task can be produced from a target grid by merely swapping the position of a horizontal element with a vertical element. (B) All possible iterations of the stimulus. Subsets were used for the masking task (black frame) and for the crowding task (white frame). The grouping task used all stimuli (rows 1-3 as targets and rows 4-5 as distractors).

The fixation mark consisted of two concentric squares. The bigger square had a side length of 0.5${}^{\circ }$and was presented either in 75% or 25% gray. The smaller square had a side length of 0.1${}^{\circ }$ and was always black. All stimuli were presented against a mid-gray background (50%, equivalent to 59.05 cd/m^2^) metameric with D65. The stimulus consisted of nine Gabor patches arranged in a 3 × 3 grid. We used this stimulus to assess masking, crowding and grouping by measuring contrast thresholds for Gabor detection, discrimination and integration, respectively. Stimulus grids can be seen in Figure 1. Gabors were created as Psychtoolbox procedural textures to ensure fast rendering. Their size was 0.8${}^{\circ }$, with a spatial frequency of 2.5 cpd and a phase-offset (± 90${}^{\circ }$) corresponding with a center-symmetric profile. Simultaneously presented Gabors always had the same randomly assigned polarity. To counteract a reduction in orientation salience with a change in stimulus contrast—either due to a reduction in perceived size when the contrast is low or due to a focus away from the orientation of the sine grating and towards the stimulus edge when the contrast is high (Henrie & Shapley, 2001)—an elliptical Gaussian envelope was used, where the major-axis of the hull was aligned with the orientation of the grating. The envelope had an aspect ratio of approximately 0.71, resulting from a standard deviation of approximately 14.3% of the patch size along the major-axis and 20% of the patch size along the minor-axis.

**Figure 2.**
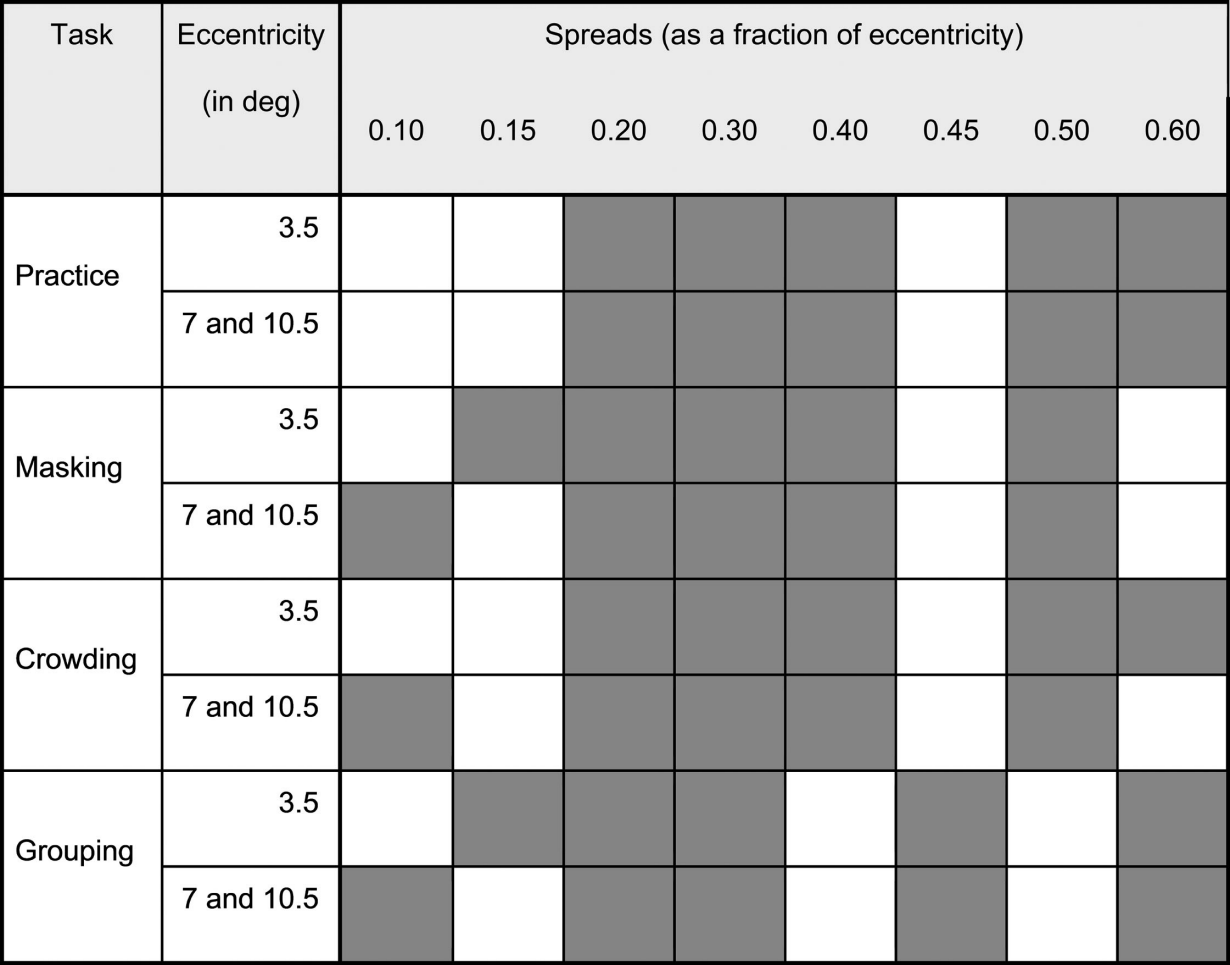
Combinations of stimulus eccentricity and spreads used in the main experiments. Spreads were chosen based on pilot data to ensure that contrast thresholds captured a range of performance levels that included both flanker-induced interference/enhancement and flanker-independent performance, while avoiding stimulus overlap in non-masking tasks. Filled (grey) boxes indicate the tested task-eccentricity-spread combinations.

Grid stimuli were presented in a spatial two alternative forced choice (2AFC) procedure with each stimulus presented equidistant on either side of a centrally presented fixation mark. Grid stimuli were presented at one of three eccentricities (3.5${}^{\circ }$, 7${}^{\circ }$, and 10.5${}^{\circ }$) with one of five spreads (Figure 2). Eccentricity refers to the distance between the centre of the fixation mark and the centre of the central Gabor of a grid stimulus while spread refers to the centre-to centre distance between individual Gabor patches along the cardinal axes (see Figure 11A, top row). The grids were centred on the horizontal mid-line. Stimulus contrast was controlled using a running-fit procedure of 40 trials. A Weibull-fit was used as the underlying psychometric curve, seeded with a standard deviation of 0.2, a slope of 3.5, a lapse rate of 0.02 and an initial threshold estimate (starting value) of 1.5-times the individual participant's average contrast for the respective stimulus eccentricity obtained in the contrast adjustment task (see section on baseline measurements). The staircase procedure was preceded by the presentation of two trials (one per staircase) with the same stimulus contrast as the starting contrast to alleviate the risk of an inattention-related lapse on the first staircase trial.

#### Masking

For the masking task, participants were presented with two grid stimuli, one on each side of fixation, and were asked to judge which one contained a central Gabor-patch. This was the target-grid. In the distractor-grid, the central Gabor was missing. The masking task used a subset of stimuli (Figure 1B, black frame), where both outer Gabor patches of the middle grid-row were either horizontal (collinear condition) or vertical (orthogonal condition). The target Gabor (central patch) was always oriented horizontally. Note that the outer Gabor patches of the middle column were the same for the collinear and the orthogonal condition (one vertical and on horizontal).

Experimental trials were blocked by spread and flanker type (orthogonal or collinear), leading to ten blocks per eccentricity. Each block consisted of two alternating interleaved staircases. Observers’ responses controlled the contrast of the stimuli in an adaptive fashion using a Weibull function (81% correct) implemented in the Palamedes toolbox. The first trial of each staircase was discarded and thus functioned as an orienting trial, giving participants a preview of the block's eccentricity and spread. This was followed by 80 trials, 40 per staircase. In this task, staircases controlled only the contrast of the central target element. The contrasts of all other Gabor-patches remained constant at 60% Michelson contrast. A light-gray fixation mark was presented before and after each trial. Participants initiated the trial via button press while maintaining central fixation. This turned the fixation mark dark-gray and brought up the stimulus with an onset asynchrony of 500 ms. The stimulus was presented for 200 ms, which is suitable to reduce the chance of eye movements to the stimulus location (e.g., Hutton, 2008; Pierce et al., 2019; Salthouse & Ellis, 1980) After an additional delay of 100 ms the fixation mark turned light-gray again to indicate that a response was expected. Participants pressed the left or right button respectively on a button-box to report the position of the target-grid. Feedback was provided in the form of beeps, where a low-pitched beep indicated a correct answer and a high-pitched beep a wrong answer. Eye-movements were monitored throughout. Trials with improper fixation were discarded online. An additional trial was added for each discarded trial. Prior to the first block, participants were presented with 60 practice trials (two per combination of eccentricity, spread and flanker orientation—collinear or orthogonal). Practice trials were presented in order of eccentricity from the closest to the furthest, and within this, from the largest to the smallest spread, and were presented with 47.5% target contrast, which should have made the task easy and given the participants a preview of the range of conditions that were to be tested.

#### Crowding

Similar to the masking task, the target in the crowding task was the central element of one of the two grid-stimuli. Here, participants were asked which of the two central Gabors was tilted to the left (target: /). The distractor-Gabor (the central Gabor in the other grid-stimulus) was tilted to the right (distractor: \). The crowding task used the same Gabor grid structure as the “collinear” stimuli of the masking task (Figure 1B, white frame), where the two lateral Gabor-patches in the middle grid-row were horizontally oriented. However, the central-patch had a tilt of $\pm 15{}^{\circ }$ relative to the horizontal axis for the target- and the distractor-Gabors, respectively. Again, trials were blocked by eccentricity and spread. Participants were presented with one block for each of the five spreads. Block structure and trial sequence were identical to those of the masking task. The same staircasing procedure was used. However, here it controlled the contrast of all Gabor elements. Participants were again presented with 60 practice trials (four per condition of eccentricity and spread). Presentation order was the same as for the practice trials of the masking task. All Gabor elements of the practice stimuli were presented with a contrast of 47.5 %. Participants reported whether the target-patch was presented in the left or the right grid-stimulus, by pressing the respective button of the button box.

#### Grouping

For the grouping task participants were again presented with two grid-stimuli. Here they were asked to indicate the presence of a line formed by three horizontally oriented Gabor elements. That is, in the target-grid, all elements in one row (top, middle or bottom) consisted of only horizontally oriented Gabors (Figure 1B, rows 1–3). In the non-target grid, a maximum of two horizontal Gabors were aligned in any one row (Figure 1B, rows 4–5). To differentiate between the target and the non-target grid, the difference in the number of aligned elements (three vs. two), makes it necessary to attend to all elements of the stimulus, especially since this difference is based on a simple position swap within the grid between one horizontally and on vertically oriented Gabor. This is facilitated further by the relatively high number of different iterations of target and non-target grids. Like the crowding task, the grouping task was blocked by eccentricity and spread, resulting in 15 blocks overall. At the outset, participants were presented with 60 practice trials (four per condition of eccentricity and spread). Practice trials were run from the smallest eccentricity and spread through the largest eccentricity and spread and were presented with 47.5% contrast. Block structure and trial sequence were identical to those of the masking task, with the only difference being that the stimulus duration was 500 ms instead of 200 ms. This duration is commonly used in studies on perceptual organisation, for example, contour integration (Mullen et al., 2000), and symmetry perception (Gheorghiu et al., 2016). Recall that fixation was monitored and hence this longer duration should not give the participants an opportunity to saccade to either grid. Stimulus contrast was controlled as in the crowding task.

The main tasks (masking, crowding and grouping) all involve a spatial 2AFC procedure where the participant is asked to indicate the location of the target grid (left or right). Traditionally, these processes have been evaluated with a one interval AFC procedure where the stimulus is presented at a single location and the participant is asked to detect, discriminate, or group elements within that stimulus, respectively. This latter method could be adapted to our stimuli to test masking and crowding while maintaining a binary response choice (present/absent or left/right tilt). However, this would not work for grouping. A line detection task (line present/absent) would not precisely capture the full extent of potential percepts. That is, since the set of three collinear Gabors (i.e., the contour) can be in the top, middle or bottom row of our stimulus, in a spatial one interval AFC task the participant would have four possible options (absent, top, bottom, middle). This would render comparisons of performance across tasks more difficult. Hence, we opted for the spatial 2AFC procedure.

### Stimuli and procedure: Baseline measurements

#### Contrast adjustment (extra-foveal)

To determine the initial contrast level for the stimuli presented in the main tasks, an adjustment task was presented on the Display++ screen. For this task, participants were presented with two horizontally oriented Gabor-patches and were asked to reduce their contrast so that they both were just noticeable simultaneously, while maintaining fixation on a centrally presented fixation mark. Gabor patches were presented twice for each of the three eccentricities (3.5${}^{\circ }$, 7.0${}^{\circ }$, or 10.5${}^{\circ }$), one on either side of the fixation mark aligned on the horizontal midline of the screen. The Gabor patches were presented with the same contrast polarity that synchronously changed every 500 ms. The specifications of the Gabor patches were the same as in the main experiments. Adjustments were made using the up and down button of the button-box. A press of the central button confirmed the adjustment.

#### Full grid detection threshold

It is possible that performance in the grouping task captured the visibility of the most peripheral Gabor-patch in the horizontal line, rather than the process of grouping the line itself. Therefore, we measured contrast detection thresholds using a spatial 2AFC procedure in which participants reported which of the two grid-stimuli, presented on the left or right side of fixation, was complete. The same stimuli as in the grouping task were used. However, here the target-grid was defined by being complete (nine Gabor patches), while for the distractor-grid one of the outermost three Gabor-patches was omitted. Participants were tested at each of the three eccentricities for the biggest spread (0.6; i.e., one block per eccentricity). Block-order was randomised. The same procedure as in the main experiment was used, including trial and block structure as well as the staircase that controlled stimulus contrast. Participants reported the target position with the button box. Participants were first presented with 30 practice trials (10 per eccentricity), ordered from the closest to the farthest eccentricity.

#### Perceived contrast matching

To assess if perceived contrast for suprathreshold stimuli differed between eccentricities, participants were instructed to adjust the contrast of one Gabor-patch (comparison) to match that of a target Gabor-patch (standard). The standard was always presented at 3.5${}^{\circ }$ eccentricity, either on the left or the right of a central fixation mark. The comparison was presented on the opposite side of the fixation mark, at an eccentricity of either 7.0${}^{\circ }$ or 10.5${}^{\circ }$. Both Gabor-patches were horizontally oriented and had the same contrast polarity. The standard patch was presented with a Michelson contrast of 0.40. Participants were presented with 12 trials per condition. For one-half of those trials the contrast of the comparison was initialised to a lower level (0.10–0.30) and for the other one-half to a higher level (0.70–0.90) than that of the standard. Eccentricity, position and contrast level of the comparison were counter-balanced; the actual initial contrast was randomly chosen within the provided range. Otherwise, the procedure was the same as for the peripheral contrast adjustment.

### Data analysis: Contrast sensitivity function

Contrast thresholds were fitted with an exponential function $\left(y\left(x\right)=\left(a+bx\right)*e^{\left(-cx\right)}\right)$ using a non-linear least squares method, where a is the y intercept of the function. From each participant's individual fit, we extracted their peak spatial frequency $\left(f_{max}=\left(c^{-1}-\frac{a}{b}\right)\right)$, their contrast threshold at peak spatial frequency$(s_{max}=\left(a+b*f_{max}e^{\left(c*f_{max}\right)}\right)$, their half bandwidth at half height in the direction of high spatial frequencies, as well as their cut-off spatial frequency for the bandwidth in this direction.

### Data analysis: Masking, crowding, and grouping

**Figure 3.**
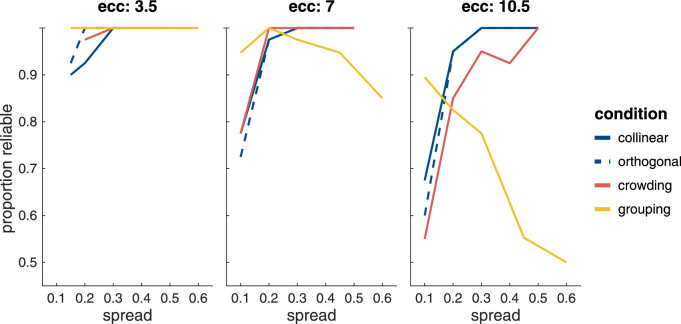
Proportion of reliably measured thresholds in the experiment. Thresholds were excluded from the analysis when the SE of the estimate exceeded 0.2.

Contrast thresholds in the main tasks are defined as the stimulus contrast at which the task performance accuracy was estimated to be 81%. These thresholds were derived by pooling the trials (*n* = 80) from the two interleaved staircases for each combination of condition, eccentricity, and spread separately and by feeding them through a running fit procedure identical to the one that controlled the contrast during stimulus presentation (Weibull function, SD 0.2, slope: 3.5, lapse rate: 0.02, initial threshold estimate set to 1.5 times the individual average contrast from the peripheral contrast sensitivity task). The reliability of the recomputed thresholds was determined using a bootstrapping procedure to ascertain the SE of the thresholds. Thresholds with a SE that exceeded 0.2 were excluded. Figure 3 shows the proportion of thresholds for each of the main tasks and conditions that fulfilled this criterion as a function of spread at each of the three eccentricities. Note that for some participants the largest spread for crowding and masking was omitted to reduce testing time if it was clear from the data that they had already reached asymptotic performance with four instead of the five inter-Gabor distances tested at that stimulus eccentricity. For masking, this was the case for two to eight participants dependent on stimulus eccentricity (four at 3.5${}^{\circ }$, eight at 7.0${}^{\circ }$, and two at 10.5${}^{\circ }$); and for crowding for zero to three (none at 3.5${}^{\circ }$, two at 7.0${}^{\circ }$, and three at 10.5${}^{\circ }$).

The resulting contrast threshold data as functions of inter-Gabor distance were then fitted with exponential functions using non-linear least square methods. Fits were derived individually for each participant, task, and eccentricity. Previously, exponential fits (1) have been used to describe accuracy data as a function of inter-object spacing (Rashal & Yeshurun, 2014; Scolari et al., 2007; Soo et al., 2018).

(1) $$\begin{array}{c} y\left(x\right)=\alpha \left(1-e^{\left(-s(x-t)\right)}\right),\; \end{array}$$

where α is the asymptote of the function, s is the scaling factor, and t is the x-intercept. Modulation of α shifts the function vertically; modulation of t shifts the function horizontally and modulation of s changes the steepness of the curve. For accuracy data, α is bound between chance and ceiling performance. To fit our contrast threshold data (*y*) as a function of inter-Gabor spacing (*x*) in degrees visual angle, we adapted the original function by inverting it:

(2) $$\begin{array}{c} y\left(x\right)=1-\alpha \left(1-e^{\left(-s(x-t)\right)}\right),\; \end{array}$$

where α and s remain the asymptote and the scaling factor of the function respectively, and t represents the x-intercept at a contrast value of 1. Parameter α was bound between 0.01 and 1. Parameters s and t were restricted to be non-negative (≥0), with an upper limit of 25 for s to avoid impossibly steep slopes. No upper limit was enforced for parameter t. From these fits we derived the baseline contrast ($y_{base}=\alpha$) that is necessary to perform the task when the inter-Gabor distance is such that it does not influence task performance. Further, we extracted the critical distance $\left(x_{crit}=t-log\left(0.1\right)/s\right)$ as the inter-Gabor distance at which the stimulus contrast is 10% higher than the baseline contrast. This is analogous to the measure used when the dependent variable is accuracy, where the critical distance is defined as the target-flanker distance at which performance is 90% of the asymptotic performance. Baseline contrast ($y_{base}$) and critical distance $\left(x_{crit}\right)$ were extracted for all fits that were considered to be reliable (${\bar{R}}^{2}\geq 0.7$) individually per participant, task and eccentricity.

The resulting data were analysed using LME models for baseline contrast and critical distance, separately. This was done for all three tasks separately and also on combined data to compare masking, crowding and grouping. LME models are advantageous for the analysis of fully balanced designs with missing data as they make row-wise exclusions unnecessary, thereby making superior use of the available data (e.g., compared with repeated-measures analysis of variance). To determine the model that best describes the data we first fitted the full model. This was either a model with one fixed factor (eccentricity) or a model with two interacting fixed factors (eccentricity and flanker orientation or task). Inter-participant variability in intercepts was included as a random effect. From this model we gradually removed first the interaction, and then the fixed factors, while assessing whether this influenced the model fit as indicated by a chi-square difference test. Only factors/interactions that influenced the model-fit were included in the final model. Follow-up pairwise comparisons were corrected for multiple comparisons (Tukey HSD) based on the number of possible comparisons (e.g., three for eccentricity). Degrees of freedom were adjusted using the Kenward-Rogers method. The LME models were run in R (version 4.0.3) using the “lme4” package (Bates et al., 2015). Pairwise differences for the estimated means were analysed using the “emmeans” package (Lenth et al., 2018). In addition, repeated measures correlations (Bakdash & Marusich, 2017) were used to analyze the relationship between baseline contrast and critical distance respectively for all tasks, as well as to analyse possible commonalities between the different tasks regarding their baseline contrasts and critical distances. Prior to these analyses, both critical distance and baseline contrast were normalized for eccentricity. This is necessary to ensure that observed correlations can be directly interpreted as correlations between the factors of interest. Thus, critical distance was expressed as a fraction of stimulus eccentricity, which is a common approach in the field (Greenwood et al., 2017). Previous studies have documented, and our current findings confirm, that contrast thresholds increase approximately linearly with eccentricity from the parafovea to the mid-periphery (Albright & Stoner, 2002; Himmelberg et al., 2020; Virsu & Rovamo, 1979). Therefore, baseline contrast was normalised by dividing the values for 3.5${}^{\circ }$, 7.0${}^{\circ }$, and 10.5${}^{\circ }$ by factors of 1, 2, and 3, respectively. Individual values were excluded from the analysis when they were at the limit of the exponential fit (i.e., baseline contrast = 0.01 before normalization) or exceeded the experimentally tested values for critical distance (i.e., >0.6). Repeated measures correlations were conducted in R using the “rmcorr” package (Bakdash & Marusich, 2017). Repeated measures correlations are superior to normal correlations when applied to data from a repeated measures design as they allow the analysis of non-aggregated data without violating the assumption of independence of observation. This does not only make better use of the available data, but also rules out several issues that arise when data from a repeated measures design are submitted to a classical correlation analysis. First, a choice needs to be made on whether correlations will be performed within each level of the repeated measures factors (e.g., eccentricity), on data averaged across levels, or on data collapsed across levels. Performing correlations separately within each level will require a large set of participants to detect anything but a strong effect, as it would necessitate correction for multiple comparisons. With a minimal repeated measures design with two factors with two levels, the criterion *p* value would already be reduced to 0.0125. In contrast, averaging the data across levels eliminates a part of the variability and reduces participants’ data to a single mean for each factor, thereby obscuring some of the individual differences (e.g., direction and magnitude of effects). Sometimes, data from several measurements is combined prior to correlational analysis without the use of summary statistics—That is, each level is entered as an independent data point. However, this leads to an inflation of the degrees of freedom and violates the assumption of independence, potentially leading to overestimations of the magnitude of correlations or (in the worst-case scenario) to false outcomes showing associations where they do not exist or associations that are incorrect in their directionality. To counteract the increased probability of a type I error as a result of testing correlations between multiple variables, we apply a more stringent alpha level of 0.01 to reject the null hypothesis.

## Results

First, we present the results of our baseline measurements and an analysis of each of the main tasks separately. This is done to evaluate whether our findings conform to predictions derived from existing literature and thus validate our experimental approach.

Second, we evaluate differences and similarities between masking, crowding and grouping using LME models and repeated measures correlations, in which we enter data from different tasks.

### Visual acuity and contrast sensitivity

**Figure 4.**
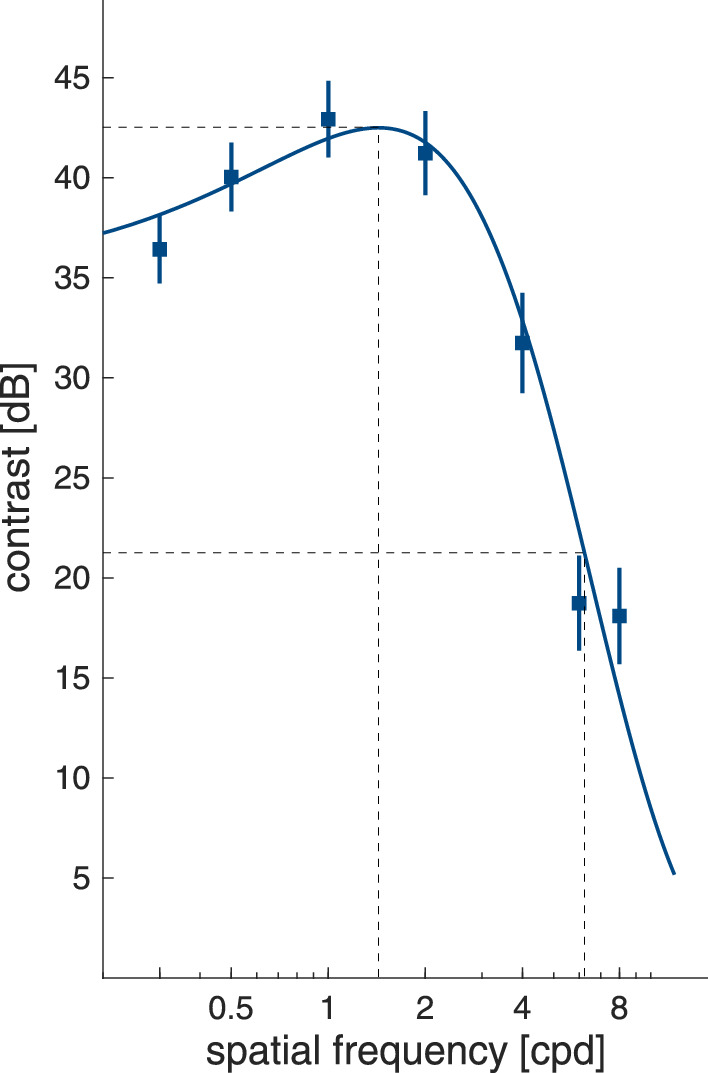
Contrast sensitivity function. Symbols show the mean contrast thresholds as a function of spatial frequency. Error bars are 95% confidence intervals. The curve shows the fit of the mean thresholds. Dashed hairlines show peak spatial frequency and peak contrast threshold, and cut-off spatial frequency for half-bandwidth at half-height, respectively.

Participants presented with a far visual acuity of −0.16 logMAR (SD 0.10) and a near visual acuity of −0.11 logMAR (SD 0.09). None of our participants failed the criterion for normal or corrected to normal vision (visual acuity of 0.04 or better) in both tests. However, two participants were slightly short of the targeted value for near (0.07 logMAR and 0.09 logMAR) and far visual acuity (0.05 logMAR both), respectively. Contrast sensitivity functions were successfully fitted (${\bar{R}}^{2}\geq 0.7$) to the data of 38 of the 40 participants (${\bar{R}}^{2}$ = 0.886 SD = 0.068). Figure 4 plots the mean CSF, revealing the expected pattern for achromatic contrast sensitivity.

**Figure 5.**
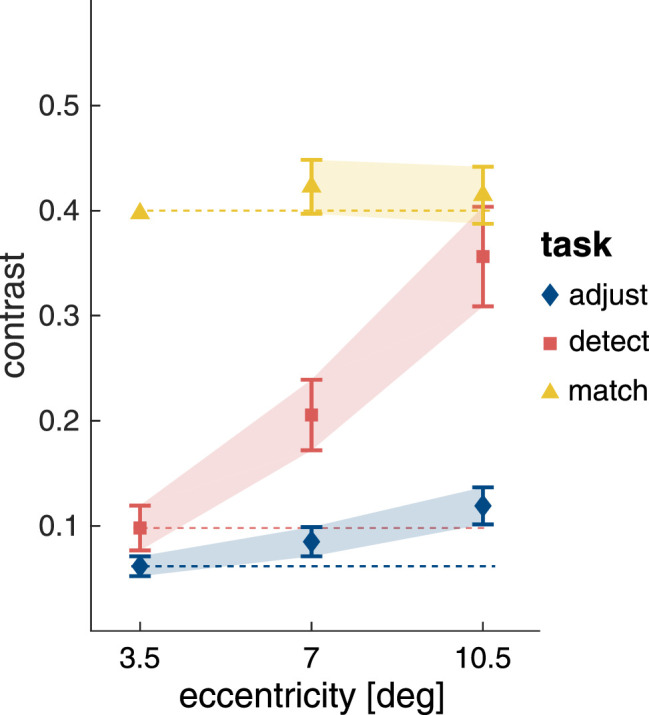
Contrast thresholds for the control measurements. Symbols show mean contrast thresholds for the adjustment, detection and matching task as functions of eccentricity, respectively. Error bars show the 95% confidence intervals. Dashed lines extend horizontally the mean at 3.5${}^{\circ }$ to ease comparisons between different eccentricities. Note that for the matching task the standard was presented at 3.5${}^{\circ }$ with a contrast of 40%.

Figure 5 shows the contrast thresholds for the contrast adjustment task, the detection task and the contrast matching task. For the contrast adjustment task, the contrast needed to detect two equidistant Gabor elements increased with eccentricity ($\chi^{2}\left(2\right)=41.9;p<.0001$), and was higher at 7.0${}^{\circ }$ compared with at 3.5${}^{\circ }$ eccentricity (t(78)=2.96;p=.011), and higher at 10.5${}^{\circ }$ compared with 7${}^{\circ }$ (t(78)=4.33;p<.001). Eccentricity also had an influence on the grid element detection task ($\chi^{2}\left(2\right)=114;p<.0001$), where participants reported which of the two grid stimuli was complete. Again, the contrast was higher at 7.0${}^{\circ }$ compared with at 3.5${}^{\circ }$ eccentricity (t(73.0)=5.93;p<.0001), and higher at 10.5${}^{\circ }$ compared to 7${}^{\circ }$ (t(73.7)=9.45;p<.0001). For the contrast matching task, no difference was observed between the contrast for the standard presented with 40% contrast at 3.5${}^{\circ }$ eccentricity and the targets presented at 7.0${}^{\circ }$ (t(74)=1.99;p=.122) and 10.5${}^{\circ }$ (t(74)=1.28;p=.412) respectively. That is, even though the contrast thresholds for detection increased with eccentricity, contrast matching at a contrast level well above the detection thresholds was not modulated by eccentricity. The results of the LME models and pairwise comparisons for these tasks are presented in Supplementary Materials 1.

These data confirm that our participants presented with visual acuity and as contrast sensitivity functions (CSFs) within the normal range. As expected, detection thresholds increase in an eccentricity-dependent fashion, while suprathreshold perceived contrast remains constant (Georgeson, 1991). The increase in detection threshold follows a largely linear pattern for both tasks, as would be expected based on how contrast sensitivity changes across the parafovea and the periphery.

### Masking

**Figure 6.**
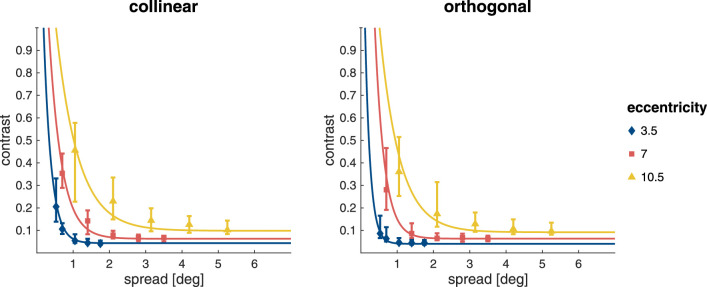
Contrast thresholds and fits for Gabor detection in the masking task. Symbols show the median contrast thresholds for a Gabor surrounded by eight distractors (60% contrast) as functions of eccentricity and inter-Gabor distance, respectively for the collinear (left) and orthogonal condition (right). Error bars show the inter-quartile range. Curves are exponential fits based on the medians of the individual participants’ fits.

**Figure 7.**
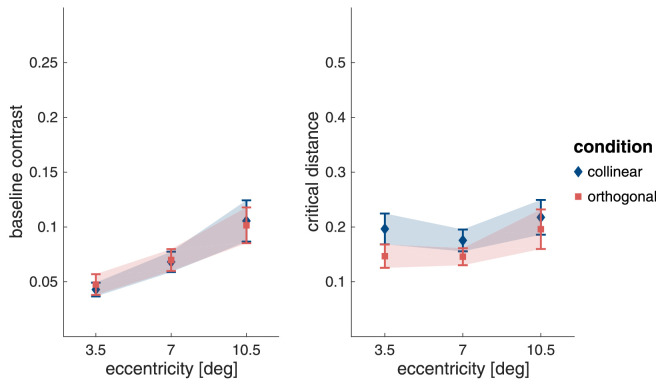
Mean baseline contrast (left panel) and mean critical distance, expressed as a fraction of eccentricity (right panel). Both show the mean with 95% confidence intervals (error bars/shaded area) for targets with collinear (blue diamonds) and orthogonal flankers (red squares).

Contrast thresholds for the masking task are shown in Figure 6 as a function of inter-Gabor spacing for both collinear and orthogonal conditions. Contrast thresholds for target detection increase with an increase in eccentricity, as well as with a decrease in inter-Gabor spacing once it drops below a critical distance. From these exponential curves, fitted to the individual participant data, we extract the baseline contrast necessary for target detection when flankers do not exert an influence, and the critical distance below which they do. The resulting mean values are shown in Figure 7.

LME models were used to assess whether baseline contrast and critical distance (as a fraction of stimulus eccentricity) were influenced by stimulus eccentricity and flanker orientation (collinear or orthogonal). The full statistical details of the best-fitting model are presented in Supplementary Materials 1. As expected, and as is evident from Figure 7, the contrast needed to detect the target outside the range of flanker influence is not influenced by flanker orientation (either independently, $\chi^{2}\left(1\right)=.120;p=.729$) or in interaction with eccentricity ($\chi^{2}\left(2\right)=.837;p=.658$). Baseline contrast increases steadily with eccentricity ($\chi^{2}\left(2\right)=132;p<.001$) and is higher for stimulus presentation at 7.0${}^{\circ }$ compared with 3.5${}^{\circ }$ eccentricity (t(137)=5.93;p<.001) and 10.5${}^{\circ }$ compared with 7.0${}^{\circ }$ eccentricity (t(137)=9.45;p<.001).

In Figure 7, critical distance appears to be slightly larger for collinear flankers compared with orthogonal flankers. The LME model analysis confirms this difference showing that collinear flankers indeed influence target detection from a farther distance than orthogonal flankers ($\chi^{2}\left(1\right)=16.0;p<.001$). Critical distance is also found to be influenced by stimulus eccentricity ($\chi^{2}\left(2\right)=22.5;p<.001$). It is higher at 10.5${}^{\circ }$ eccentricity than at 3.5${}^{\circ }$ (t(138)=3.57;p=.001) and at 7.0${}^{\circ }(t\left(137\right)=4.76;p<.0001$) eccentricity. There is no difference between critical distances at 3.5${}^{\circ }$ and 7.0${}^{\circ }$ (t(137)=-1.10;p=.517) eccentricity. Eccentricity and flanker condition do not interact ($\chi^{2}\left(2\right)=2.19;p=.334$).

**Figure 8.**
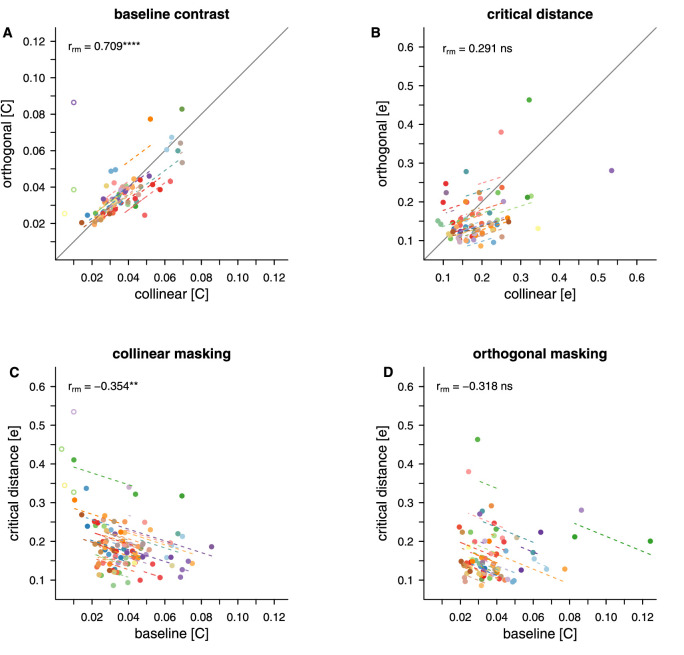
Repeated measures correlations. Correlations are shown between the two flanker conditions of the masking task for normalised baseline contrast (A), and critical distance as a fraction of stimulus eccentricity (e), and between normalised baseline contrast and critical distance for both flanker conditions, respectively (C and D). Dots indicate individual thresholds. Matching colours within a plot indicate that the thresholds stem from the same participant. Dashed lines show the correlation for individual participants over two to three threshold pairs. Data points were excluded from the analysis (empty circles) when their value was based on the limit of the exponential fit (baseline contrast = 0.01 before normalisation) or exceeded the presented values for critical distance (>0.6).

Figure 8 visualizes the results of the repeated measures correlations between the collinear and orthogonal flanker conditions for baseline contrast and critical distance (both normalised for eccentricity), as well for the baseline contrast and critical distance data for each of the flanker conditions. Baseline contrast is strongly correlated for collinear and orthogonal flanker orientations ($r_{rm}$ (33) = 0.709; *p* < 0.0001). There is no evidence of a robust correlation between critical distances in collinear and orthogonal flanker conditions ($r_{rm}$ (34) = 0.291; *p* = .086). However, even though the intra-individual patterns of differences are not consistent enough across the three eccentricities to provide a significant correlation, the distribution of the critical distance data in Figure 8B is in line with the LME model, according to which collinear flankers influence target detection from a farther distance than orthogonal flankers. This is indicated by the majority of the data points falling below the line of equality and a positive slope clearly below 1. Finally, critical distance is found to be negatively correlated with baseline contrast in the collinear flanker condition ($r_{rm}$(55) = −0.354; *p* = .007). That is, the lower the contrast threshold for target detection when it is not influenced by flankers, the bigger the distance from which an influence is observed. For the orthogonal flanker condition ($r_{rm}$(42) = −0.318; *p* = .036) we do not observe a similarly stable link between baseline contrast and critical distance.

While the baseline contrast at which elements can be detected without flanker interference depends majorly and solely on eccentricity, critical distance also depends on the flanker type, being smaller for orthogonally oriented flankers. This confirms that we successfully targeted different extents of spatial interference using grids with orthogonal and iso-oriented flankers. Baseline contrast is strongly driven by retinal eccentricity, as manifested also by robust correlations across the tested locations for differently oriented flankers. Meanwhile, critical distance behaves more idiosyncratically. The fact that we can robustly identify the expected patterns of retinal co-dependence in terms of baseline contrast shows that the lack of clear patterns for critical distance is not due to a lack of power, but more likely due to the distinctiveness of the interference windows across the tested retinal locations. We also observe a link between critical distance and the contrast bottleneck: when contrast detection thresholds are low, even far away flankers affect performance, while for higher baseline contrast thresholds, flankers have to be closer to enact an influence.

### Crowding

**Figure 9.**
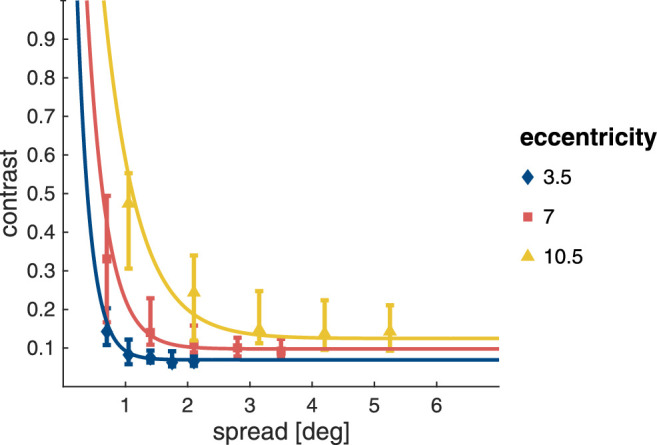
Contrast thresholds and fits for orientation discrimination in the crowding task. Symbols show median contrast thresholds, while error bars show the interquartile range.

Figure 9 shows the contrast thresholds and exponential fits for each of the combinations of target eccentricity and inter-Gabor distance. Like the detection thresholds in the masking task, contrast thresholds for target discrimination in the crowding task seem to increase with an increase in stimulus eccentricity, as well as with a decrease in inter-Gabor spacing once it drops below a critical distance.

**Figure 10.**
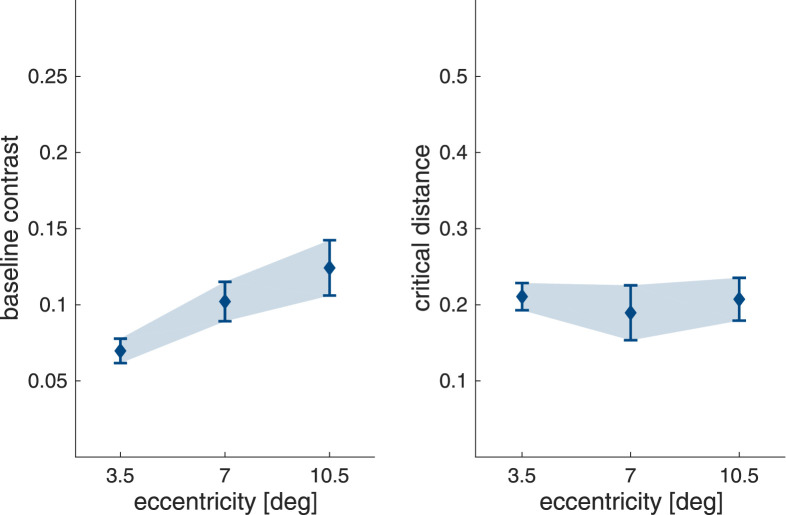
Baseline contrast and critical distance as a fraction of target eccentricity for the crowding task. Both panels show means (symbols) with 95% confidence intervals (error bars/shaded area).

The mean values for baseline contrast and critical distance extracted from the exponential fits to the individual participant data are shown in Figure 10. Again, LME models were used to assess whether baseline contrast and critical distance (as a fraction of stimulus eccentricity) were influenced by stimulus eccentricity.

As can be seen in Figure 10A, the baseline contrast for target discrimination increased with eccentricity ($\chi^{2}\left(2\right)=53.2;p<.001$), and was higher at 7.0${}^{\circ }$ compared with 3.5${}^{\circ }$ eccentricity (t(50.3)=578;p<.001), and for 10.5${}^{\circ }$ compared with 7.0${}^{\circ }$ eccentricity ((52.8)=4.55;p<.001). The critical distance of crowding was also modulated by stimulus eccentricity ($\chi^{2}\left(2\right)=8.89;p=.012$). While there was no difference in critical distance for stimulus presentation at 3.5${}^{\circ }$ and 10.5${}^{\circ }$ eccentricity ((50.4)=0.525;p=.859), the critical distance at 7.0${}^{\circ }$ eccentricity was somewhat smaller compared with both (vs. 3.5${}^{\circ }$: (49.7)=2.76;p=.022; vs. 10.5${}^{\circ }$: t(51.7)=3.02;p=.011). The full statistical details are given in Supplementary Materials 1.

**Figure 11.**
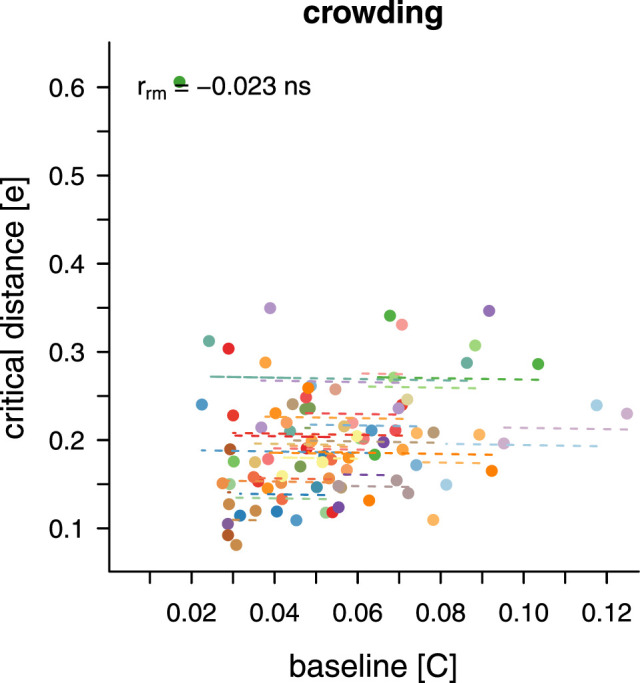
Repeated measures correlations. Correlations are shown between baseline contrast and critical distance for the crowding task. Dots indicate individual thresholds. Matching colours indicate that the thresholds stem from the same participant. Dashed lines show the correlation for individual participants over two to three threshold pairs.

To explore the relationship between baseline contrast and critical distance in the crowding task the values extracted from the exponential fits were also submitted to a repeated measures correlation (see Figure 11). No correlation was found ($r_{rm}$(48) = −0.023; *p* = .874).

Unsurprisingly, the baseline contrast in the absence of flanker interference, here for orientation discrimination, is again strongly modulated by eccentricity. More unexpectedly, for critical distance we also find a very small but statistically robust modulation, with a slightly narrower interference window at 7.0${}^{\circ }$. Regardless, critical distance remains relatively flat between 3.5${}^{\circ }$ and 10.5${}^{\circ }$, resulting in a lack of correlation with baseline contrast, which robustly increases with eccentricity. This is markedly different from masking, where a link between baseline contrast and critical distance can be observed.

### Grouping

**Figure 12.**
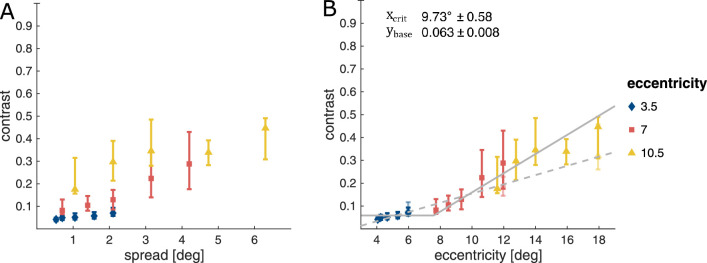
Contrast thresholds in the grouping task. (A) The thresholds as a function of inter-Gabor distance. (B) The same data as a function of eccentricity of the most peripheral Gabor element. Data is fitted with a clipped line fit (solid line) and presented against the detection thresholds (faint symbols at 6${}^{\circ }$, 12${}^{\circ }$ and 18${}^{\circ }$) with a linear fit (dashed line). Symbols show the median contrast thresholds for the stimulus grid. Error bars show the inter-quartile range.

Figure 12 shows the contrast thresholds for the grouping task. From Figure 12A, it is obvious that, as was observed in the masking and crowding task, contrast thresholds increase with eccentricity. Thresholds appear to be lowest when the grid stimulus was presented at 3.5${}^{\circ }$, higher when presented at 7.0${}^{\circ }$, and even higher when presented at 10.5${}^{\circ }$. Contrast thresholds also seem to be modulated by inter-Gabor distance, where contrast thresholds increase with an increase in the inter-Gabor spacing. We had expected to see the inverse of the effect of inter-Gabor spacing on contrast thresholds for masking and crowding. That is, for each of the three stimulus eccentricities, one would have predicted contrast thresholds to be asymptotic when the Gabor-elements were reasonably close, and to increase markedly once they exceeded a critical distance. This would be in line with the concept of an association field with a size that scales with eccentricity, where elements that fall within it are readily combined, whereas elements that fall outside the limits of the association field avoid being subjected to integration. However, rather than finding this pattern individually for each of the three stimulus eccentricities, we found very little contrast threshold modulation at 3.5${}^{\circ }$. Whereas, when the stimulus was centred at 7.0${}^{\circ }$ and 10.5${}^{\circ }$, respectively, contrast thresholds increased with an increase in inter-Gabor distance, without exhibiting a lower asymptote.

Figure 12B replots the contrast thresholds as a function of the eccentricity of the farthest Gabor element. Given our task and stimulus, participants would need to identify the orientation of the outermost Gabor elements to successfully integrate them with the closer elements, to in turn correctly report the target, since grouping, unlike masking and crowding, relies on integration of several elements, rather than on the individuation and detection/identification of a single element. Therefore, rather than being dependent on the eccentricity of the centre of a stimulus object, grouping might be dependent on the identification of the furthest stimulus element. Hence, we replotted the original data shown in Figure 12A as a function of the eccentricity of the farthest Gabor element over all three stimulus eccentricities (Figure 12B). Of course, the eccentricity from fixation to the outermost Gabors differs between the top and bottom rows of the stimulus grid and the middle row of the grid. Therefore, the data and analysis presented in Figure 12B are computed over contrast thresholds estimated from the data for the three-aligned-Gabor targets only in the top and bottom rows.

As outlined above, we computed a clipped line fit for each of the 40 participants, which resulted in a reliable fit (${\bar{R}}^{2}\geq 0.7$) for 34 of them, with a mean ${\bar{R}}^{2}$ of 0.847 (SD = 0.073) for the successful fits. This can be taken to indicate that integration in our experiment indeed was stable over a relatively large area as indicated by the asymptotic part of the fitted function, and that contrast thresholds outside of this area showed a slight increase with an increase in element eccentricity, as indicated by the rising part of the fitted function with a median slope of 0.042 per degree (interquartile range, 0.033–0.065). From these fits, we extracted a single value for the *baseline contrast* that is necessary for feature integration (asymptote) and a single value for the *critical eccentricity*, as the distance at which stimulus contrast is 10% higher than baseline contrast.

**Figure 13.**
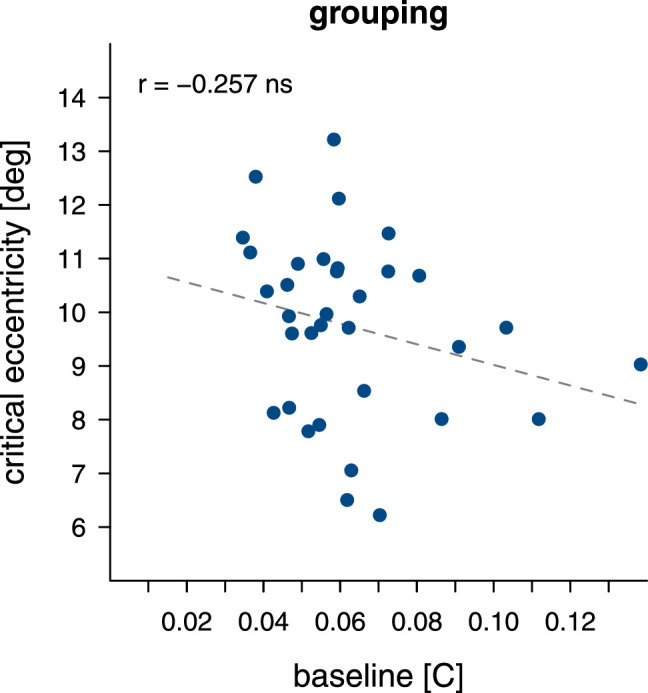
Correlation between baseline contrast and the critical distance values extracted from clipped line fits to the individual participant data. Each dot represents the data of a single participant.

In our sample, we observe a mean baseline contrast threshold of 0.063 ± 0.008 and a critical eccentricity of 9.73${}^{\circ }$ ± 0.58. The individual participant data is shown in Figure 13. We do not find evidence of a robust correlation between baseline contrast and critical eccentricity (r(32)=-0.257;p=0.089).

**Figure 14.**
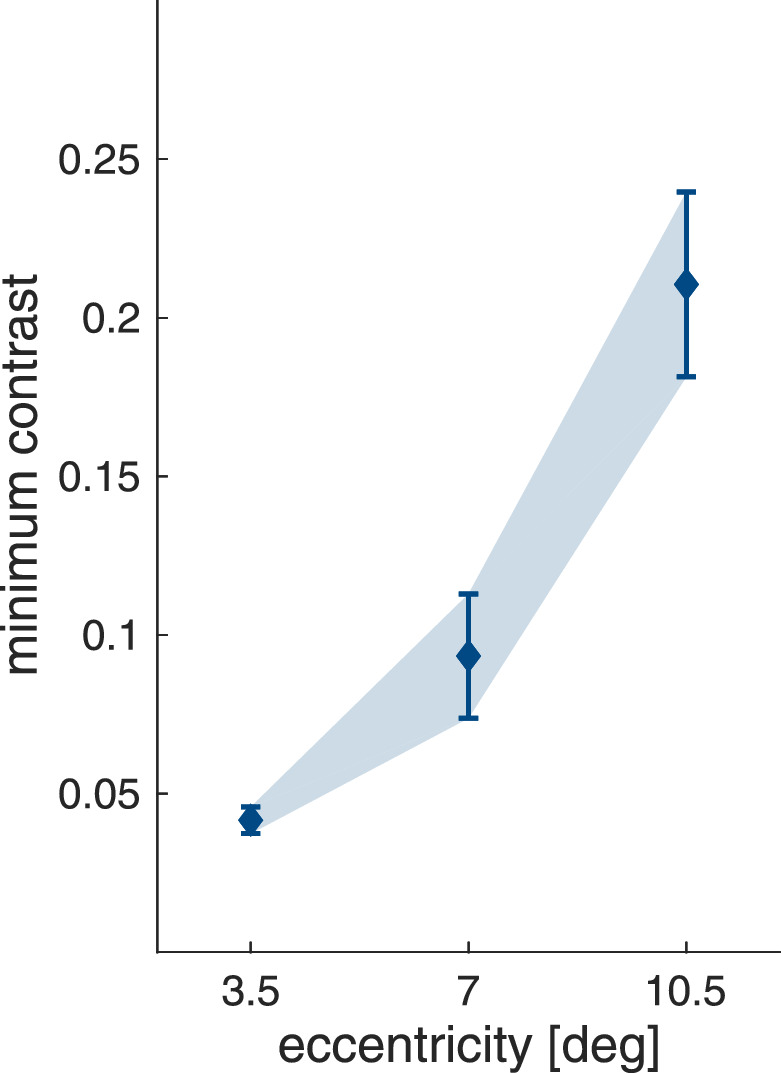
Minimum contrast for the grouping task as a function of stimulus eccentricities (mean ±95% confidence interval).

For masking and crowding, we were able to directly assess the influence of stimulus eccentricity on baseline contrast for target detection and discrimination, respectively. To allow a comparable analysis for contour integration, we also extracted the *minimum contrast* for each of the three stimulus eccentricities (3.5${}^{\circ }$, 7.0${}^{\circ }$ and 10.5${}^{\circ }$) at which performance was estimated to be 81%. As can be observed in Figure 14 and was confirmed by a LME model ($\chi^{2}\left(2\right)=103;p<.001$), the minimum contrast necessary for feature integration was influenced by stimulus eccentricity and was found

to be higher at 7.0° eccentricity compared with 3.5${}^{\circ }$ (t(73.2)=4.28; p<.001), and at 10.5${}^{\circ }$ compared with 7.0${}^{\circ }$ (t(72.8)=9.48;p<.001).

To summarize the findings of the grouping task, rather than finding measurable windows of facilitation by grouping at each of the three tested eccentricities, we discover that our data conforms more easily to a “broken stick” function dependent on the eccentricity of the entire stimulus (defined by the furthest element), with one value for baseline contrast and critical distance. That is, we observe an initially, relatively low level of contrast that only starts to increase beyond a distance of 10${}^{\circ }$. We like to point out that this function is not a simple product of the increase in detection threshold for all the grid elements. Detection contrast increases more linearly, so that for the closest distances grouping contrasts fall at or below detection threshold, while for the furthest stimuli they reliably surpass it.

### Comparisons between masking, crowding, and grouping

#### Differences between masking, crowding, and grouping

**Figure 15.**
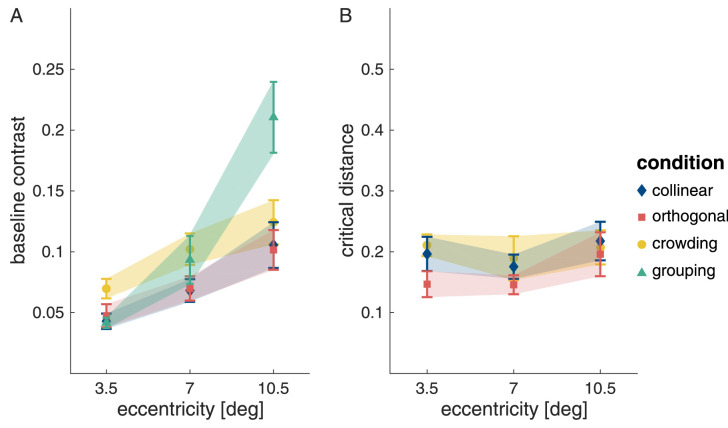
Baseline contrast and critical distance as a fraction of target eccentricity. (A and B) The means (symbols) with 95% confidence intervals (error bars/shaded area). Note that for grouping the graph shows the minimum contrast rather than the baseline contrast. Also, we could not measure critical distance for grouping but rather ended up with a measure of critical eccentricity, thus, grouping is absent from (B).

The separate analyses of the baseline contrast thresholds and critical distances for masking and crowding, and of the minimum contrast thresholds for grouping all showed an expected influence of eccentricity. The extracted values are replotted in Figure 15 for comparison. To evaluate how these tasks may differ, we first compared the baseline contrast thresholds for masking and crowding. Since the separate analyses had not shown a difference in the baseline contrast thresholds between the two flanker-orientation conditions in the masking task, crowding was compared to the mean of the collinear and orthogonal flanker conditions. A LME model confirmed the observation that baseline contrasts were higher in the crowding task compared with the masking task ($\chi^{2}\left(2\right)=65.9;p<.001;$ for more detail, see Supplementary Material 1). There was no interaction of task with eccentricity ($\chi^{2}\left(4\right)=1.80;p=.773$).

Next, we compared critical distances (Figure 15B) and found that they also differed across these two tasks. Here crowding was compared with the two flanker conditions of the masking task separately, since our previous analysis indicated that critical distance was higher for collinear compared with orthogonal flankers. Critical distance for crowding was higher than critical distance for masking with orthogonal flankers (t(224)=4.09;p=<.001), whereas there was no difference in the critical distance between crowding and masking with collinear flankers (t(222)=0.317;p=.946). Again, there was no interaction between task and eccentricity ($\chi^{2}\left(4\right)=5.37;p=.251$).

Finally, we compared the minimum contrast for element integration in the grouping task with the baseline contrast for target detection in the masking task (mean of collinear and orthogonal flanker conditions) and with the baseline contrast for target orientation discrimination in the crowding task. The LME model confirmed that both eccentricity and task modulated contrast thresholds. The two factors were also found to interact ($\chi^{2}\left(6\right)=109;p<.001$). Figure 15A shows that the minimum contrast required for element integration in the grouping task increases more steeply with eccentricity than baseline contrast in the masking and crowding tasks. At the closest eccentricity, contrast thresholds for the grouping task did not differ from those of the masking task (t(329)=-0.679;p=.873) but were lower than those of the crowding task (t(329)=-3.09;p=.007). At 7${}^{\circ }$ eccentricity, the thresholds for the grouping task exceed those of the masking task (t(328)=3.10;p=.006), but were not different from the threshold for the crowding task (t(328)=-0.865;p=.770). At the farthest stimulus eccentricity, the thresholds for the grouping task exceeded both masking and crowding thresholds (ts>8.81;ps<.001). The full LME model results and post-hoc pairwise comparisons can be seen in Supplementary Materials 1.

The steeper increase in contrast for the grouping task is to be expected. This task requires the processing of three Gabor elements, which at close distances occurs close to detection threshold but at further distances requires roughly twice the contrast necessary for detection and discrimination of single element properties. The finding that the critical window for crowding was no different than for masking with collinear flankers was somewhat unexpected, although we do observe the expected smaller critical window for orthogonal flankers.

#### Similarities between masking, crowding, and grouping

**Figure 16.**
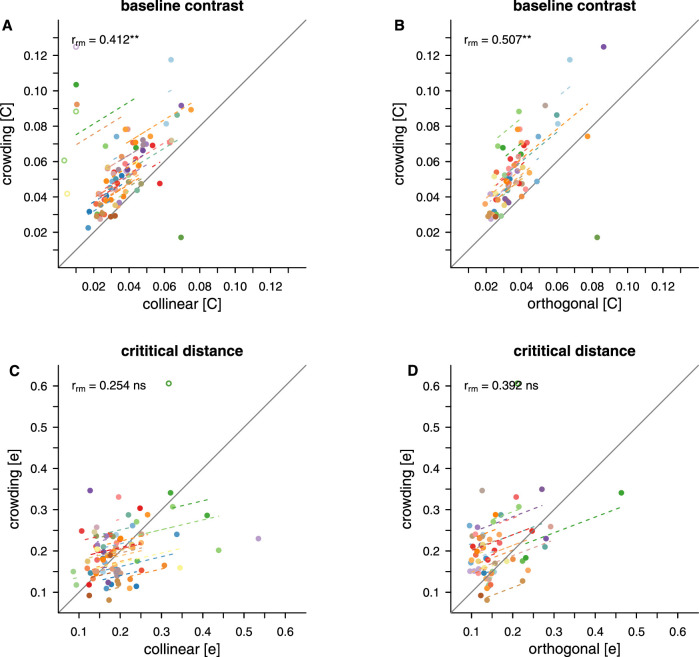
Repeated measures correlations for masking and crowding Correlations are shown between crowding and both flanker conditions of the masking task for baseline contrast (A and B) and for critical distance (C and D). Dots indicate individual thresholds. Matching colours indicate that the thresholds stem from the same participant. Dashed lines show the correlation for individual participants over two to three data pairs. Data points were excluded from the analysis (empty circles) when their value was at the limit of the exponential fit (baseline contrast = 0.01) or exceeded the presented values for critical distance (>0.6).

To further explore the relationships between the different tasks and conditions, and to test whether baseline contrast and critical distances are associated between tasks, repeated measures correlations were conducted. Again, we first assessed the relationship between masking and crowding. Figure 16 shows the correlations between crowding and the two flanker conditions of the masking task. Baseline contrast for crowding had a moderate positive correlation with both collinear ($r_{rm}\left(34\right)=0.412;p=.009$) and orthogonal ($r_{rm}\left(30\right)=0.507;p=.004$) flanker masking conditions. That is, stimuli presented to retinal locations that needed higher contrast for target detection in the masking task also required higher contrast for orientation discrimination in the crowding task. Overall, the plots also demonstrate that baseline contrast for crowding was higher than baseline contrast for masking for the majority of participants. In contrast, robust correlations were not found between the critical distance for crowding and critical distance for masking with either collinear ($r_{rm}\left(34\right)=0.254;p=.104$) or orthogonal flankers ($r_{rm}\left(34\right)=0.392;p=.032$).

**Figure 17.**
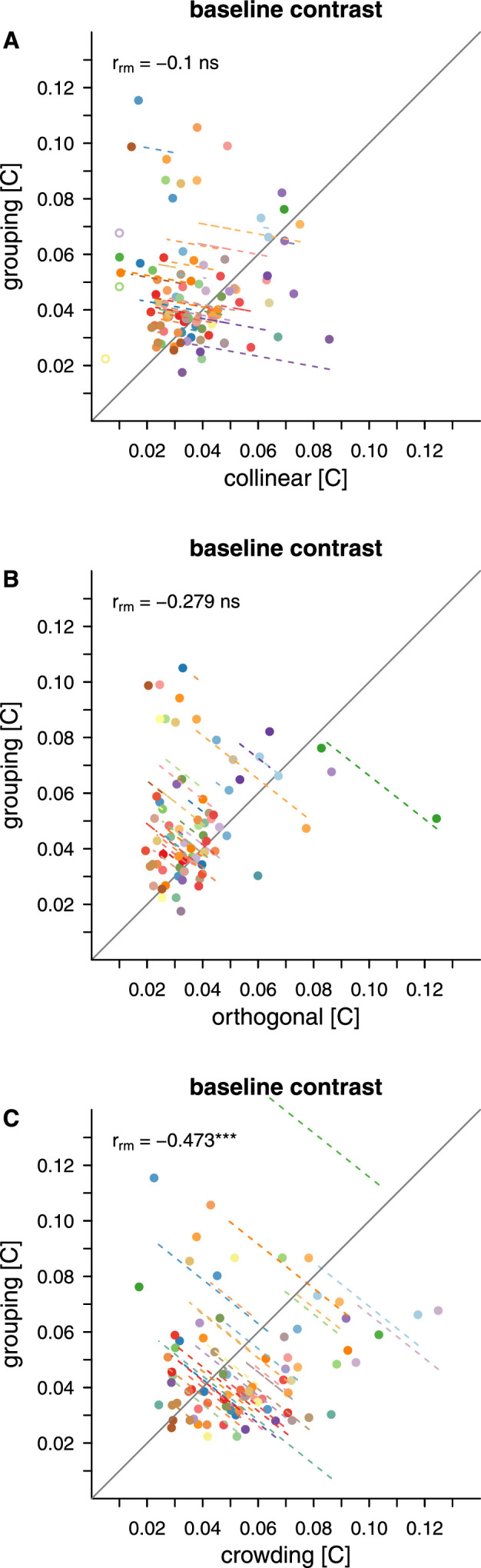
Repeated measures correlations for grouping Correlations are shown between grouping and the two flanker conditions of the masking task (A and B) and crowding (C). The dots indicate individual thresholds. Matching colours indicate that the thresholds stem from the same participant. Dashed lines show the correlation for individual participants over two to three data pairs. Note that to allow for a repeated-measures analysis across eccentricities we use baseline contrast thresholds for masking and crowding, while for grouping we use minimum contrast. For masking and crowding, data points were excluded from the analysis (empty circles) when their value was at the limit of the exponential fit (baseline contrast = 0.01).

Repeated measures correlations between minimum contrast for contour integration in the grouping task and the two flanker types in the masking task, and with the crowding task are shown in Figure 17A–C. While there was insufficient evidence of a reliable correlation between grouping and masking with collinear flankers ($r_{rm}\left(36\right)=-0.100;p=.474$) and between grouping and masking with orthogonal flankers ($r_{rm}\left(33\right)=-0.279;p=.073$), the minimum contrast for grouping was found to be negatively correlated with baseline contrast for crowding ($r_{rm}\left(36\right)=-0.473;p=.001$). The higher the contrast needed for orientation discrimination of the target in the crowding task, the lower the contrast needed to integrate elements in the grouping task.

Our repeated measures correlations confirm commonalities in contrast dependencies between detection and discrimination, on one hand, and discrimination and integration, on the other hand. However, we find no links among the tasks in terms of critical distances. This is unsurprising; critical distances for crowding remain roughly stable across the tested retinal locations, unlike those for masking which depend on contrast.

Perhaps most interestingly, we find that detection and discrimination benefit from contrast in a similar way, while discrimination and integration in an inverse way. These relations mimic the previously described similarities between masking and crowding (Lev & Polat, 2015) and disjunctions between crowding and grouping (Chakravarthi & Pelli, 2011). The novelty of our finding is that our threshold approach reveals these links at the level of contrast required to perform the three respective tasks, before any flanker influence is even manifest.

## Discussion

To assess commonalities and differences between low and mid-level vision processes that detect, discriminate and integrate spatially distinct visual elements, we measured contrast thresholds for masking, crowding and grouping. We evaluated all three processes using a common test pattern at a series of locations in the parafovea and the near periphery for a range of inter-element distances. This allows us to characterize each process when faced with a requirement to perform its diagnostic task (detect, discriminate, integrate).

Our approach involved determining the baseline contrast and critical distance for each of the tasks. Baseline contrast indicates the contrast required to do the respective diagnostic task without notable flanker interference (masking and crowding) or hampered integration (grouping). The critical distance indicates the spatial extent over which this contrast threshold is stable and beyond which a change in inter-element distance leads to threshold elevation over baseline. While the baseline contrast needed to drive reliable task performance allows conclusions about the visual processes underlying detection, discrimination and integration, the spatial extent allows assertions about the putative visual field that determines whether elements are readily combined or not, which is favourable for contour integration but unfavourable for tasks relying on element individuation.

**Figure 18.**
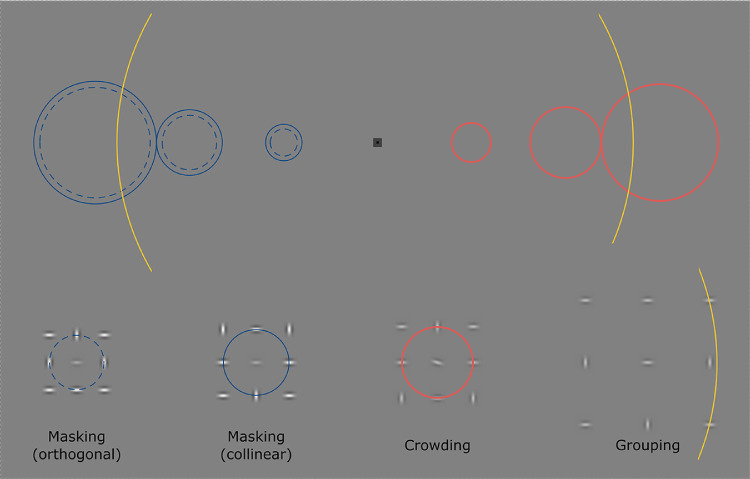
Visualization of the spatial extent over which elements are combined. (Top) Spatial windows are depicted relative to the position of the fixation cross and the three tested retinal locations. Masking spatial windows are depicted in blue (dashed – orthogonal masking; full line – collinear masking), crowding windows in red and the window for grouping in yellow. (Bottom) Spatial windows are depicted over the stimulus grid, to give a better idea of the size relative to the position of the grid elements.

Our investigation reveals the following properties under the constraints imposed by our nine-element grid stimulus. The baseline contrast needed to perform the diagnostic task increases with eccentricity, with a steeper increase for feature integration, compared to feature detection and feature discrimination. Comparing low- and mid-level visual processes, feature discrimination requires 2.8 percentage points more contrast than feature detection overall, while feature integration requires more contrast only in the near periphery (2.4 percentage points at 7.0${}^{\circ }$, but 10.7 percentage points more at 10.5${}^{\circ }$ eccentricity). In the parafovea, contrast thresholds for feature integration did not exceed those for feature detection. The critical distance for masking and crowding was found to scale with eccentricity. The orthogonal flanker condition of the masking task yielded the smallest scaling factor (approximately 17% of the eccentricity). Collinear flankers influenced target detection from a slightly farther distance with a scaling factor that was similar to the one we observed for target discrimination in the crowding task (approximately 20% of the eccentricity). On the other hand, instead of being dependent on the inter-Gabor distance as a function of stimulus eccentricity, the contrast threshold for grouping was better described as a function of eccentricity of entire stimulus defined by the distance of the farthest Gabor element from fixation. That is, we observed a relatively stable contrast threshold for contour integration stimuli that fell within approximately $10{}^{\circ }$ from the fovea. To illustrate these findings and facilitate comparisons, spatial areas over which each process is active (critical distance) are depicted in Figure 18.

### Contrast as a key bottleneck of stimulus processing

Unsurprisingly, contrast thresholds were found to be higher for tasks targeting mid-level vision compared to tasks targeting low-level vision. Uniform positive correlations between the contrast thresholds for the diagnostic tasks (detect, discriminate, integrate) could suggest a trivial link, driven by a task-independent, basic constraint that does not have anything to do with long-range spatial interactions.^1^ However, controlling for a common association with eccentricity, by normalising the data for a general increase in contrast threshold with an increase in eccentricity (Himmelberg et al., 2020; Virsu & Rovamo, 1979), we find a more complex pattern of results. While baseline contrasts for detecting stimuli and discriminating their properties remain positively linked across the tested retinal locations, integrating elements and discriminating their properties are negatively linked. The latter finding extends previous reports of an inverse relationship between crowding and contour integration (Chakravarthi & Pelli, 2011; May & Hess, 2007). However, a similarly robust link between contrast needed for detection and integration is lacking. Even after normalisation, factors that lead to an increase in detection thresholds also lead to an increase in discrimination thresholds. Conversely, factors that lead to an increase in integration thresholds lead to a reduction in discrimination thresholds.

Such a pattern of baseline contrast results that relies on the spatial requirements of the respective tasks should not be surprising. After all, contrast does influence receptive field size, with increases by up to 2.3 to 2.5 times under conditions of low contrast (2.5: Cavanaugh et al., 2002; 2.3: Sceniak et al., 1999). This increase can be successfully modelled through divisive inputs into a centre/surround field; at low contrasts, the surround would become relatively weak and thereby exert reduced suppression, leading to the expansion of the centre (Cavanaugh et al., 2002). Confirming these observations, our repeated measures correlations reveal that reduced baseline contrast corresponds with an increased spatial window of interference for collinear flankers. However, we fail to observe a similarly robust relation for the discrimination task, most likely due to a relatively fixed nature of the integration field, whose spatial extent remains relatively stable (here, roughly 20% of eccentricity), irrespective of stimulus contrast.

One criticism of our approach may be that in manipulating contrast within a threshold framework, we are favoring an outcome that reveals contrast-dependent bottlenecks. A typical alternative would be an experimental framework in which contrast is fixed to a supra-threshold value and the effect of element proximity is captured using behavioral measures (e.g., percent correct, as in Greenwood et al., 2017). The risk with this approach, however, is that it may be biased away from common constraints that originate from a contrast-bound, low-level process such as masking. Any conclusions would then be less generalizable, as they would only capture operational parameters at a fixed, high level of contrast, rather than across a range of contrasts that enable threshold performance. Neri (2018) proposes a fully specified, mechanistic model of vision based on a combination of simple-like and complex-like units, in which early architectural constraints may be shared between various feature-processing requirements. In line with our experimental and interpretive logic, the emergence of a contrast-dependent bottleneck is therefore informative about a more fundamental characteristic of the visual system, namely, that contrast detection and discrimination, on one hand, and discrimination and integration, on the other hand, share some of the same circuitry and thus exhibit similar contrast dependencies with eccentricity. Meanwhile, spatial windows remain uncorrelated across eccentricity, suggesting that the computations performed by each type of visual field (perceptual, integrative and associative), in terms of linking elements across space, operate over different distances that are idiosyncratic to the specific task. In contrast, when data are aggregated and between-participant correlations are assessed (see Supplementary Materials 3), individual difference patterns in spatial interactions in line with Greenwood et al. (2017) are observed: participants who have a smaller spatial window for masking also have a smaller spatial window for crowding, and vice versa.

### Critical distance and visual field size

Visual fields determine the spatial extent of information that is processed within different neural units in the visual cortex. While information that falls within the same visual field is bound together, information that falls into different visual fields is individuated. In this study, we used critical distance to estimate the area over which contrast thresholds are modulated by inter-element distance.

For the masking task, the critical distance was found to be modulated by flanker orientation: collinear flankers exerted an influence on target detection from a farther distance than did orthogonal flankers. This is in line with the idea that collinear flankers influence detection of a target element when they fall into neighbouring fields, while orthogonal flankers need to encroach on the same visual field to exert an effect (Lev & Polat, 2011; Polat & Sagi, 1993). Using critical distance from orthogonal flanker masking as an estimate of field size, we find the perceptive field size to be 0.6${}^{\circ }$ at 3.5${}^{\circ }$, 1.2${}^{\circ }$ at 7.0${}^{\circ }$ and 1.8${}^{\circ }$ at 10.5${}^{\circ }$. While our values are overall lower compared with those observed by Lev and Polat (2011), with a doubling of perceptive field size estimated for 7.0${}^{\circ }$ compared with 3.5${}^{\circ }$ eccentricity, our study also confirms a magnification factor of 2 reported in previous studies (Klein et al., 1990; Mäkelä et al., 1993; Rovamo & Virsu, 1979). The overall lower estimate is likely the result of a difference in the method used to estimate field size. Methods that focus on the facilitatory effect of flanker elements or on the transition between facilitatory and suppressive effects are likely to yield higher estimates. Using orthogonal masking as an estimate is likely to reveal the center of the perceptive field instead, for which suppression is observed when flanker elements fall in the same spatial unit as the target. While spatial 2AFC paradigms, as the one used in our study, control for criterion shifts to report a target as present, they fail to highlight a change from facilitation to suppression (Giorgi et al., 2004; Williams & Hess, 1998; Zenger-Landolt & Koch, 2001). Also, the presentation of additional flankers alongside the collinear triplet would have further reduced the chance of observing facilitatory effects as iso-oriented, non-collinear flankers (e.g., presented side by side with the target, here above or below) had previously been shown to forestall such modulation (Lev & Polat, 2016; Solomon & Morgan, 2000), while additional more randomly distributed and randomly oriented Gabor patches do not pose such an obstacle (Polat & Bonneh, 2000).

The critical distance over which flanking elements interfere with target discrimination in crowding tasks has previously been used to estimate the size of the integration field (e.g., Pelli, 2008; Pelli et al., 2004; Pelli & Tillman, 2008; Poder, 2006; Yeshurun & Rashal, 2010). We observed integration field sizes of 0.7${}^{\circ }$ at 3.5${}^{\circ }$, 1.4${}^{\circ }$ at 7.0${}^{\circ }$ and 2.1${}^{\circ }$ at 10.5${}^{\circ }$ and hence a scaling factor of 0.2 or 20% of the eccentricity. The scaling factor observed here is comparatively low in contrast with the 40% to 50% of the target eccentricity usually expected in crowding paradigms (Bouma, 1970; Pelli et al., 2004; Scolari et al., 2007; Toet & Levi, 1992), but see Strasburger (2020a, 2020b). However, it lies within the range that is normally reported (13%–80%; e.g., Chung et al., 2001; Soo et al., 2018; Strasburger & Malania, 2013); also see (Pelli et al., 2004). Interestingly, the critical distance for crowding did not exceed the critical distance for collinear masking. The near correspondence in their sizes is somewhat surprising given that crowding is thought to occur over large distances and masking is supposed to be a more local phenomenon. One could argue that the observed similarity in field sizes and scaling with eccentricity for the two processes could coincidentally be driven by the similarity of our stimulus set up. The stimulus configuration for the two tasks was the same apart from two factors: the contrast of the flanker elements (fixed at 60% for masking, variable with target contrast for crowding), and the orientation of the target element (0${}^{\circ }$ for masking, ±15${}^{\circ }$ for crowding). Yet based on the relatively broad tuning of orientation-sensitive neural elements (10–25${}^{\circ }$; Campbell & Kulikowski, 1966; Movshon & Blakemore, 1973; Thomas & Gille, 1979), it is unlikely that feature-tuned detectors could readily drive performance in the crowding task. Rather than being based on the excitation of the most excited neural element, narrowly tuned orientation discrimination relies on the comparison in excitation of elements that differ in orientation tuning (Regan & Beverley, 1985). Alternatively, the low contrast of all elements and the low confusability of the target (oblique target tilt compared with horizontal and vertical flankers) might have contributed to the overall small interaction window for crowding, leading to the low difference in field size between masking and crowding.

Superficially, there seems to be a link between the spatial extents for masking and crowding. However, this is primarily because critical spacings for both tasks scale with eccentricity. Indeed, the linear mixed effect models showed that this scaling factor is relatively stable across eccentricities. Hence field sizes for both will appear to be co-dependent, even within an observer (as one grows the other grows). Controlling for this trivial dependence on eccentricity eliminates any robust correlation between the two. Therefore, the repeated measures correlations (or rather a lack of them) can be interpreted as showing no evidence that the residual noise that remains after scaling is in some way correlated. In other words, the contrast detection mechanism operates on a different spatial scale in the presence of flanking information compared with the contrast discrimination mechanism. This might be taken to suggest that they are implemented by neural populations that pool information using different mechanisms. For example, we might speculate that masking occurs within fields of simple-like neural units with centre-surround architecture, whereas crowding occurs within fields of complex-like units that have centers without surrounds but that pool information as a weighted sum of nearby detected features (Harrison & Bex, 2015; Van den Berg et al., 2010; Yashar et al., 2019). While the two processes might be contrast linked, as discussed in the previous section, the spatial interactions they carry out, and hence the distance over which these interactions occur, are distinct and shaped by the specific task they implement (detection versus discrimination). This is in line with the findings by Greenwood et al. (2017) who argued that although multiple visual processes appear to share similar spatial constraints, this does not indicate shared spatial representations but instead demonstrates the idiosyncratic spatial topology inherited across these processes.

The size of the grouping-related association fields proved more difficult to capture. Unlike for masking and crowding where we were able to estimate critical distance per stimulus eccentricity, grouping was instead found to depend on the eccentricity of the furthest away Gabor element. That is, we observed a relatively stable contrast threshold for contour integration stimuli that fell within 10${}^{\circ }$ from the fovea. This is consistent with Hess and Dakin's (1997) model, in which a simple contrast-driven process can perform contour grouping up to 10° of eccentricity (but also see Nugent et al., 2003). In other words, the size of the association field, as captured by our task, does not seem to scale with eccentricity - unlike the signatures we captured for perceptive and integrative fields.

This supports the idea that there is a degree of disjunction between processes underlying crowding and grouping. Previous studies have argued that integrative and association fields are the same or at least related (Chakravarthi & Pelli, 2011; May & Hess, 2007). Similarly, grouping processes have been thought to be tightly linked to crowding (Herzog et al., 2015; Livne & Sagi, 2007, 2010). While it might be the case that grouping processes affect crowding, our findings point to the possibility that the underlying mechanisms are distinct. It is important to keep in mind at least one difference between masking/crowding and grouping tasks. To successfully complete the former tasks, the aim has to be to narrow attention to a specific and relatively small region of space (at the target's location), whereas for completing a grouping task, the aim would be to be spread out attention more. This difference might drive some of the observed differences between the tasks. In contrast, our findings do reveal some links between the two phenomena, but they rather stem from a common contrast bottleneck: the variation of contrast thresholds for feature integration and feature discrimination across eccentricities were negatively correlated.

## Future directions

### Modeling

It would be useful to build a computational model that could account for a contrast bottleneck that shares commonalities across retinal locations between masking, crowding and grouping. This model could be informed by computational efforts in the field of texture perception, as these models already incorporate both suppressive and facilitatory influences. Wilkinson, Wilson and Ellemberg (1997) assessed flanker interference for target elements that belonged to a parafoveally presented texture (2° to 6° eccentricity). Similar to our findings, the presence of surround elements induced marked threshold elevations that increased in strength as inter-element spacing decreased and as retinal eccentricity increased. Wilkinson and colleagues accounted for the data with a model with simple and complex cells that had somewhat different sensitivities and were in a network with reciprocal interactions. Activity of complex cells suppressed that of simple cells. The contrast processing model of Neri (2018) also discusses the relative contributions of simple and complex cells to various aspects of low and high-contrast driven task performance. It would be interesting to build a model of featurally tuned simple and complex cells and evaluate how this system performs in terms of detection, discrimination and integration across a range of contrasts, inter-element spacings and eccentricities. This would allow us to address several important questions. One such question would concern the emergence of crowding at relatively low contrasts. In a recent study, we found that oriented flankers began to enact a graded influence on performance only once the orientation of a Gabor could be reliably discriminated (i.e., only when the stimuli were suprathreshold). Meanwhile, in participants for whom the same stimuli were very close to orientation discrimination threshold, we observed a relatively strong flanker interference that was invariant to distance from target (Lee et al., 2021). With an effective computational model, we can systematically evaluate discrimination performance across the low-to-mid contrast range and observe if similar behaviour emerges, with the prediction being that complex cell pooling at contrast levels that do not allow precise featural extraction should result in a spacing-invariant interference rather than spacing-sensitive impairment due to flankers. Note that, however, some recent models of impaired discrimination (crowding) incorporate suppressive surrounds in their simulated neural populations to account for interference between visual elements with multiple features (Greenwood & Parsons, 2020). It would be interesting to determine the conditions under which the different receptive field architectures become important in these processes. Finally, Greenwood et al. (2017) suggest that retinotopic cell density and receptive field size are key spatial drivers of between-observer idiosyncrasies. The proposed computational model could evaluate the consequences of spatial sampling density by simple cells on both masking and crowding, but this question could also be experimentally pursued through the use of adaptive optics (see e.g., Kwon & Liu, 2019).

### Psychophysics

In our study, we found that both masking and crowding could be relatively well described regarding the parameters of interest, namely, baseline contrasts and spatial extents. However, the spatial extent for the grouping task was harder to capture. Our results indicate that grouping seems to be stable within a 10° radius around the fovea. Nevertheless, a part of the reason why the spatial extent is hard to capture might be due to the stimulus. Generally speaking, in stimuli used to test grouping, it is notoriously hard to simultaneously control for multiple factors, such as eccentricity, inter-element distance and display density without creating artefacts or confounds. In the current study we were constrained by having to create a stimulus that is basically the same for all three tasks. Therefore, we were unable to completely disentangle the influence of inter-element distance and element eccentricity, which co-varied. Second, it is not obvious that aligning three horizontal elements sufficiently captures grouping processes.

In a follow-up study, we are addressing these issues by a) using a stimulus display that more closely resembles a typical contour integration task (“snake in the grass”) with multiple elements aligned within the contour and b) varying the inter-element distance with all contour elements presented at the same eccentricity (Reuther et al. V-VSS, 2021). With such a setup, we are measuring contrast thresholds for contour integration as functions of eccentricity and inter-element spacing while closely controlling for influences of display density. This would draw a more comprehensive picture of grouping in the parafovea and near periphery while further testing the conclusions drawn in this study about grouping.

## Conclusion

From a theoretical perspective, it is important to understand the degree of separability of neural mechanisms that underlie spatial interactions in low and mid-level vision. The advantage of our approach is that we used the same paradigm to investigate three underlying processes (masking, crowding and grouping) and the same conceptual level of analysis (visual field type) to frame our findings. We found that the main processing constraint that is shared between progressively more complex visual fields that receive, integrate and associate information reside more firmly within the units that process contrast, rather than within putative separate units that determine their spatial windows of interaction. This is in line with the general finding that the size of receptive fields depends on contrast. Greenwood et al. (2017) propose that visual field idiosyncrasies stemming from receptive field sizes and retinotopic cell densities are inherited across many tasks and that there is no shared spatial representation. Contrast-processing units may thus lie at the core of the shared idiosyncrasies reported in many previous studies, despite the fundamental differences in the extent of their spatial windows.

## Supplementary Material

Supplement 1

Supplement 2

Supplement 3

## Footnotes

1 Analysis for the raw (non-normalized) data is presented in the Supplementary Materials 2.
