# Supplementary material for: Masking, crowding, and grouping: Connecting low and mid-level vision
Source: J Vis. 2022 Feb 11;22(2):7. doi: 10.1167/jov.22.2.7 (PMC8842520; doi:10.1167/jov.22.2.7)
Supplement: Supplement 1 [file jovi-22-2-7_s001.docx]

# Supplementary material 1 – Results for the best fitting LME models

In addition to the results of the LME models reported in the main manuscript, the tables report the estimated means $\pm$ SE, as well as $\Omega^{2}$ for the best fitting model.

### Baseline measures

Supplementary Table 1.1: Best fitting LME models for the baseline measures

| **adjusted contrast** ~ eccentricity + (1\|participant) | | | | | $\Omega^{2}= .607$ | |
| --- | --- | --- | --- | --- | --- | --- |
| Significant factors | Estimates ± SE | | Pairwise comparisons | | | |
| eccentricity  $\chi^{2} \left( 2 \right)=41.9$ $p<.0001$ | 3.5°: | $0.062\pm.007$ | 3.5° vs 7°: | $t_{\left( 78 \right)}=2.96$ | | $p=.011$ |
|  | 7°: | $0.085\pm.007$ | 7° vs 10.5°: | $t_{\left( 78 \right)}=4.33$ | | $p<.001$ |
|  | 10.5: | $0.119\pm.007$ |  |  | |  |
| **detection contrast** ~ eccentricity + (1\|participant) | | | | | $\Omega^{2}= .854$ | |
| Significant factors | Estimates ± SE | | Pairwise comparisons | | | |
| eccentricity  $\chi^{2} \left( 2 \right)=114$ $p<.0001$ | 3.5°: | $0.098\pm.017$ | 3.5° vs 7°: | $t_{\left( 73.0 \right)}=5.93$ | | $p<.0001$ |
|  | 7°: | $0.206\pm.017$ | 7° vs 10.5°: | $t_{\left( 73.7 \right)}=9.45$ | | $p<.0001$ |
|  | 10.5: | $0.367\pm.018$ |  |  | |  |
| **matching contrast** ~ 1 + (1\|participant) | | | | | $\Omega^{2}= .544$ | |
| Tested factor | Estimates ± SE | | Pairwise comparisons | | | |
| eccentricity  $\chi^{2} \left( 2 \right)=4.06$ $p=.132$ | 3.5°: | $0.400\pm.011$ |  |  | |  |
|  | 7°: | $0.423\pm.011$ | 3.5° vs 7°: | $t_{\left( 74 \right)}=1.99$ | | $p=.122$ |
|  | 10.5: | $0.415\pm.011$ | 3.5° vs 10.5°: | $t_{\left( 74 \right)}=1.28$ | | $p=.412$ |

Note that for the contrast masking task the contrast for the standard (0.40) was submitted to the model for the eccentricity of 3.5°

### Masking

Supplementary Table 1.2: Best fitting LME models for baseline contrast and critical distance in the masking task

| **baseline contrast** ~ eccentricity + (1\|participant) | | | | | | $\Omega^{2}= .771$ | |
| --- | --- | --- | --- | --- | --- | --- | --- |
| Significant factors | Estimates ± SE | | Pairwise comparisons | | | | |
| eccentricity  $\chi^{2} \left( 2 \right)=132$ $p<.0001$ | 3.5°: | $0.046\pm.005$ | 3.5° vs 7°: | $t_{\left( 137 \right)}=5.93$ | | | $p<.0001$ |
|  | 7°: | $0.069\pm.005$ | 7° vs 10.5°: | $t_{\left( 137 \right)}=9.45$ | | | $p<.0001$ |
|  | 10.5: | $0.107\pm.005$ |  |  | | |  |
| **critical distance** ~ flanker orientation + eccentricity + (1\|participant) | | | | | $\Omega^{2}= .582$ | | |
| Significant factors | Estimates ± SE | | Pairwise comparisons | | | | |
| flanker orientation  $\chi^{2} \left( 1 \right)=16.0$ $p<.0001$ | collinear: | $0.203\pm.010$ | coll. vs orth.: | $t_{\left( 138 \right)}=4.08$ | | | $p=.0001$ |
|  | orthogonal: | $0.170\pm.010$ |  |  | | |  |
| eccentricity  $\chi^{2} \left( 2 \right)=22.5$ $p<.0001$ | 3.5°: | $0.178\pm.011$ | 3.5° vs 7°: | $t_{\left( 137 \right)}=-1.10$ | | | $p=.517$ |
|  | 7°: | $0.167\pm.010$ | 7° vs 10.5°: | $t_{(137)}=4.76$ | | | $p<.0001$ |
|  | 10.5: | $0.214\pm.011$ | 3.5° vs 10.5°: | $t_{\left( 138 \right)}=3.57$ | | | $p=.001$ |

### Crowding

Supplementary Table 1.3: Best fitting LME models for baseline contrast and critical distance in the crowding task

| **baseline contrast** ~ eccentricity + (1\|participant) | | | | | $\Omega^{2}=.824$ | |
| --- | --- | --- | --- | --- | --- | --- |
| Significant factors | Estimates ± SE | | Pairwise comparisons | | | |
| eccentricity  $\chi^{2} \left( 2 \right)=53.2 p<.0001$ | 3.5°: | $0.068\pm.006$ | 3.5° vs 7°: | $t_{\left( 50.3 \right)}=5.78$ | | $p<.0001$ |
|  | 7°: | $0.100\pm.006$ | 7° vs 10.5°: | $t_{\left( 52.8 \right)}=4.55$ | | $p=.0001$ |
|  | 10.5: | $0.128\pm.007$ |  |  | |  |
| **Critical distance** ~ eccentricity + (1\|participant) | | | | | $\Omega^{2}= .757$ | |
| Significant factors | Estimates ± SE | | Pairwise comparisons | | | |
| eccentricity  $\chi^{2} \left( 2 \right)=8.89$ $p=.012$ | 3.5°: | $0.219\pm.015$ | 3.5° vs 7°: | $t_{\left( 49.7 \right)}=-2.76$ | | $p=.022$ |
|  | 7°: | $0.185\pm.015$ | 7° vs 10.5°: | $t_{\left( 51.7 \right)}=3.02$ | | $p=.011$ |
|  | 10.5: | $0.226\pm.016$ | 3.5° vs 10.5°: | $t_{(50.4)}=.525$ | | $p=.859$ |

### Grouping

Supplementary Table 1.4: Best fitting LME model for the minimum contrast in the grouping task

| **minimum contrast** ~ eccentricity + (1\|participant) | | | | | $\Omega^{2}=.701$ | |
| --- | --- | --- | --- | --- | --- | --- |
| Significant factors | Estimates ± SE | | Pairwise comparisons | | | |
| eccentricity  $\chi^{2} \left( 2 \right)=103 p<.0001$ | 3.5°: | $0.042\pm.009$ | 3.5° vs 7°: | $t_{\left( 73.2 \right)}=4.28$ | | $p=.0002$ |
|  | 7°: | $0.093\pm.009$ | 7° vs 10.5°: | $t_{\left( 72.9 \right)}=9.48$ | | $p<.0001$ |
|  | 10.5: | $0.213\pm.010$ |  |  | |  |

### Differences between masking and crowding

Supplementary Table 1.5: Best fitting LME models comparing masking and crowding.

| **baseline contrast** ~ task + eccentricity + (1\|participant) | | | | | $\Omega^{2}= .709$ | |
| --- | --- | --- | --- | --- | --- | --- |
| Significant factors | Estimates ± SE | | Pairwise comparisons | | | |
| task  $\chi^{2} \left( 2 \right)=65.9$ $p<.0001$ | masking: | $0.074\pm.004$ | mas. vs crow.: | $t_{\left( 221 \right)}=8.63$ | | $p<.0001$ |
|  | crowding: | $0.102\pm.005$ |  |  | |  |
| eccentricity  $\chi^{2} \left( 2 \right)=168$ $p<.0001$ | 3.5°: | $0.055\pm.004$ | 3.5° vs 7°: | $t_{\left( 221 \right)}=7.23$ | | $p<.0001$ |
|  | 7°: | $0.080\pm.004$ | 7° vs 10.5°: | $t_{(221)}=9.25$ | | $p<.0001$ |
|  | 10.5: | $0.115\pm.005$ | 3.5° vs 10.5°: |  | |  |
| **critical distance** ~ task + eccentricity + (1\|participant) | | | | | $\Omega^{2}= .452$ | |
| Significant factors | Estimates ± SE | | Pairwise comparisons | | | |
| task  $\chi^{2} \left( 2 \right)=20.2$ $p<.0001$ | crowding: | $0.205\pm.010$ | crow. vs coll.: | $t_{\left( 222 \right)}=.317$ | | $p=.946$ |
|  | collinear: | $0.202\pm.009$ | crow. vs orth.: | $t_{\left( 224 \right)}=4.09$ | | $p=.0002$ |
|  | orthogonal: | $0.166\pm.010$ |  |  | |  |
| eccentricity  $\chi^{2} \left( 2 \right)=19.5$ $p<.0001$ | 3.5°: | $0.188\pm.010$ | 3.5° vs 7°: | $t_{\left( 222 \right)}=-1.78$ | | $p=.178$ |
|  | 7°: | $0.182\pm.009$ | 7° vs 10.5°: | $t_{(223)}=4.49$ | | $p<.0001$ |
|  | 10.5: | $0.213\pm.011$ | 3.5° vs 10.5°: | $t_{\left( 224 \right)}=2.70$ | | $p=.020$ |

For baseline contrast and the factor task the pairwise comparison was set up to compare crowding to the mean of collinear and orthogonal masking (Helmert regression style coding).

### Differences between masking, crowding and grouping

Supplementary Table 1.6: Best fitting LME models comparing grouping with masking and crowding.

| baseline contrast | | | | $\Omega^{2}= .683$ | | | |
| --- | --- | --- | --- | --- | --- | --- | --- |
| Fixed factors | Estimates ± SE | | | | Pairwise comparisons | | |
| eccentricity by task  $\chi^{2} \left( 6 \right)=109$  $p<.0001$ | 3.5° | masking | $0.047\pm.008$ | | grouping |  |  |
|  |  | crowding | $0.070\pm.008$ | | vs. masking | $t_{\left( 329 \right)}=-.679$ | $p=.873$ |
|  |  | grouping | $0.042\pm.007$ | | vs. crowding | $t_{\left( 329 \right)}=-3.09$ | $p=.007$ |
|  | 7° | masking | $0.070\pm.007$ | | grouping |  |  |
|  |  | crowding | $0.101\pm.007$ | | vs. masking | $t_{\left( 328 \right)}=3.10$ | $p=.006$ |
|  |  | grouping | $0.093\pm.007$ | | vs. crowding | $t_{\left( 328 \right)}=-.865$ | $p=.770$ |
|  | 10.5° | masking | $0.106\pm.008$ | | grouping |  |  |
|  |  | crowding | $0.129\pm.008$ | | vs. masking | $t_{\left( 331 \right)}=13.1$ | $p<.0001$ |
|  |  | grouping | $0.213\pm.007$ | | vs. crowding | $t_{\left( 331 \right)}=8.41$ | $p<.0001$ |

Contrasts were set up to allow comparison between masking (mean of collinear and orthogonal), crowding and grouping (user-defined regression coding).
