# Supplementary material for: Masking, crowding, and grouping: Connecting low and mid-level vision
Source: J Vis. 2022 Feb 11;22(2):7. doi: 10.1167/jov.22.2.7 (PMC8842520; doi:10.1167/jov.22.2.7)
Supplement: Supplement 2 [file jovi-22-2-7_s002.docx]

# Supplementary material 2 – Comparisons between Correlations on Raw and Normalised Contrast Data

To examine the pairwise relationships between the baseline contrasts for masking, crowding, and grouping we used a repeated measures correlation analysis. For each participant, 2 to 3 data pairs were entered into the analysis. Repeated measures correlations allow conclusions to be drawn about the relationship of two variables, here baseline contrast of two tasks, rather than about their variability across participants. Within-participant patterns of variability that emerge consistently across the three eccentricities (3.5, 7 and 10.5 degrees) could be taken as being in favour of processing commonalities that operate across the tested retinal locations.

For the analysis presented in the manuscript, we normalised the data to control for a general increase in the contrast threshold with eccentricity. Contrast thresholds have been shown to increase linearly between parafoveal locations and the mid-periphery (Himmelberg et al., 2020; Rovamo & Virsu, 1979). This increase is observed for all our tasks (see the linear mixed effect model analyses in the manuscript). Supplementary Figure 2.1 shows the baseline contrast for all tasks as a function of eccentricity – the increase with eccentricity is clearly observed for the majority of the participants. Analysing such data with repeated measures correlations runs the risk that any observed relationship stems from the simple fact of decreasing contrast sensitivity with increasing eccentricity in all tasks. Indeed, as can be seen from Supplementary Table 2.1, strong positive correlations are manifest in the raw data due to this trivial connection.

Supplementary Figure 2.2 shows the same data depicted in Supplementary Figure 2.1 after normalisation. The masking data now shows no obvious modulation with eccentricity, as indicated by a relatively flat slope. Meanwhile, crowding and grouping show modulations that go beyond the basic increase in contrast with eccentricity. Importantly, both masking conditions are still strongly correlated, indicating that any residual variation beyond the increase with eccentricity is shared. As repeated-measures correlations are unaffected by linear scaling of the data, any links that remain after normalisation can therefore be interpreted as processing commonalities that operate across the tested retinal locations. For example, in Supplementary Figure 2.1, minimum contrast for grouping has a noticeably steeper slope than baseline contrast for the crowding task, meaning that at parafoveal retinal locations less contrast is needed to integrate Gabor elements than to discriminate their orientation. This relationship reverses in the periphery. This connection is maintained in the normalised data and exhibits a clear within-participant correlation, with a negative correlation indicating opposite contrast requirements across retinal locations in the two tasks.

Supplementary Table 2.1: Repeated measures correlation analysis for the raw and normalised baseline contrast data

|  |  |  |  | raw | | | | Normalised | | | |
| --- | --- | --- | --- | --- | --- | --- | --- | --- | --- | --- | --- |
|  |  |  | n | r | p | inter | slope | r | p | inter | slope |
| baseline | collinear | orthogonal | 30 | .878 | <.0001 | -.001 | .997 | .709 | <.0001 | .009 | .746 |
|  | collinear | crowding | 34 | .896 | <.0001 | .029 | .937 | .412 | .009 | .031 | .616 |
|  | orthogonal | crowding | 30 | .779 | <.0001 | .048 | .715 | .507 | .004 | .023 | .824 |
|  | collinear | grouping | 36 | .720 | <.0001 | -.020 | 1.68 | -.100 | .474 | .056 | -.182 |
|  | orthogonal | grouping | 33 | .634 | <.0001 | -.016 | 1.81 | -.279 | .073 | .087 | -.783 |
|  | crowding | grouping | 36 | .627 | <.0001 | -.070 | 1.81 | -.473 | .001 | .094 | -.794 |


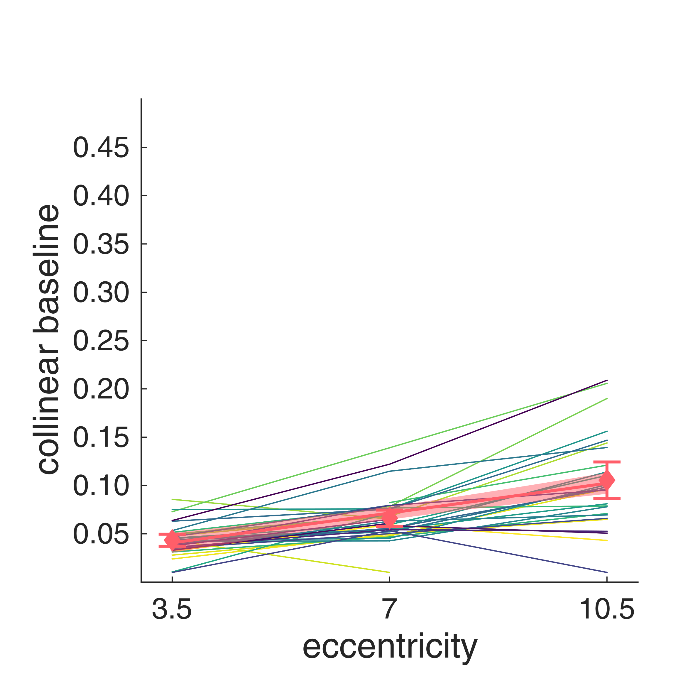

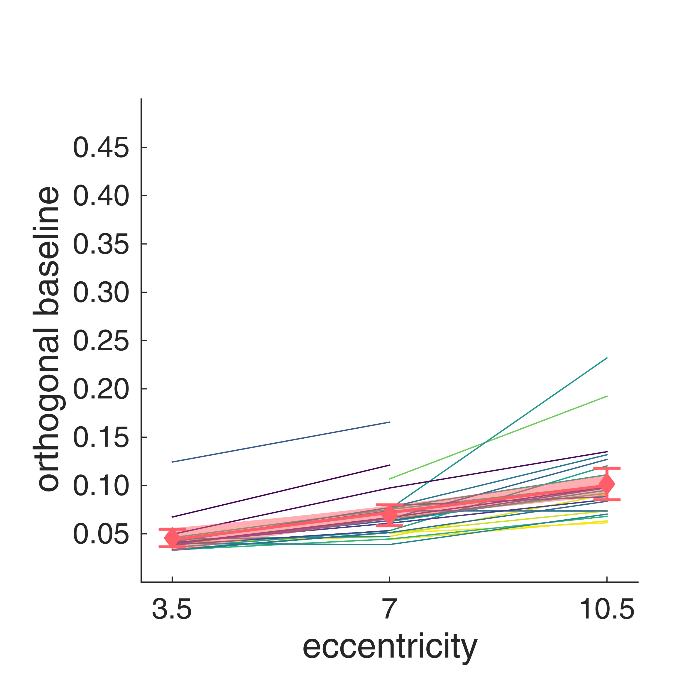


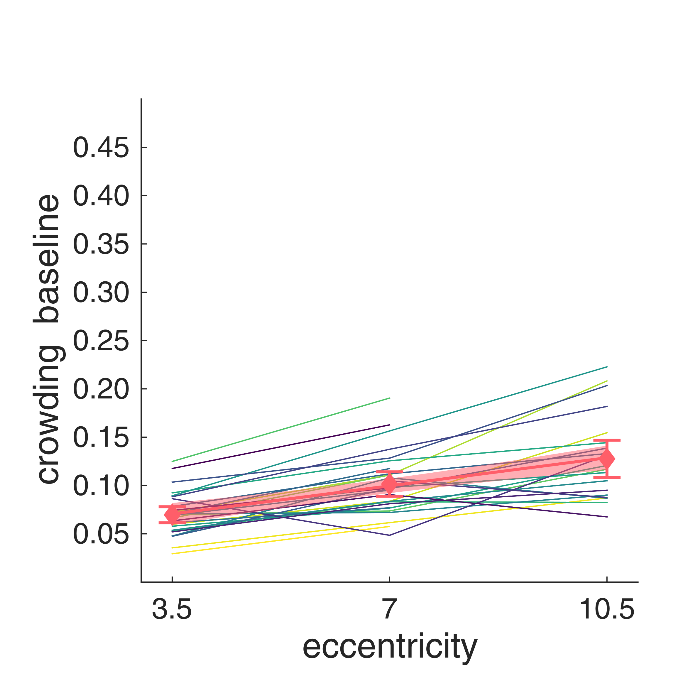

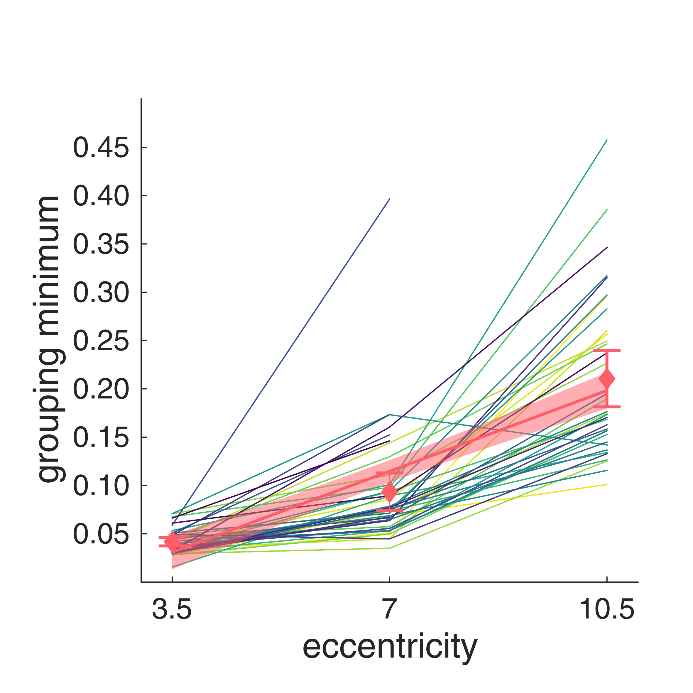


Supplementary Figure 2.1: Baseline contrast as a function of eccentricity for each of the main tasks. Individual participant data is plotted (each participant represented by line with a unique colour) alongside the mean per eccentricity (diamonds with error bars: 95% CI) and a general linear model (pink line with a shaded 95% CI).


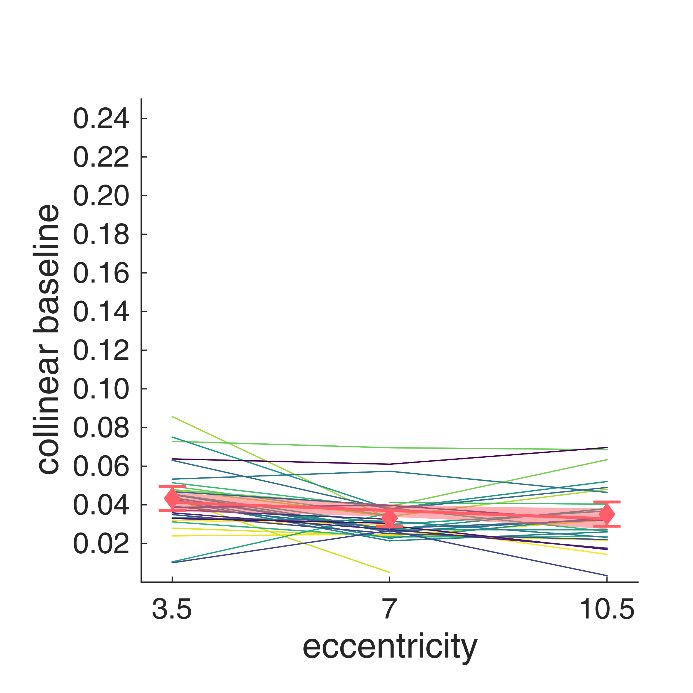

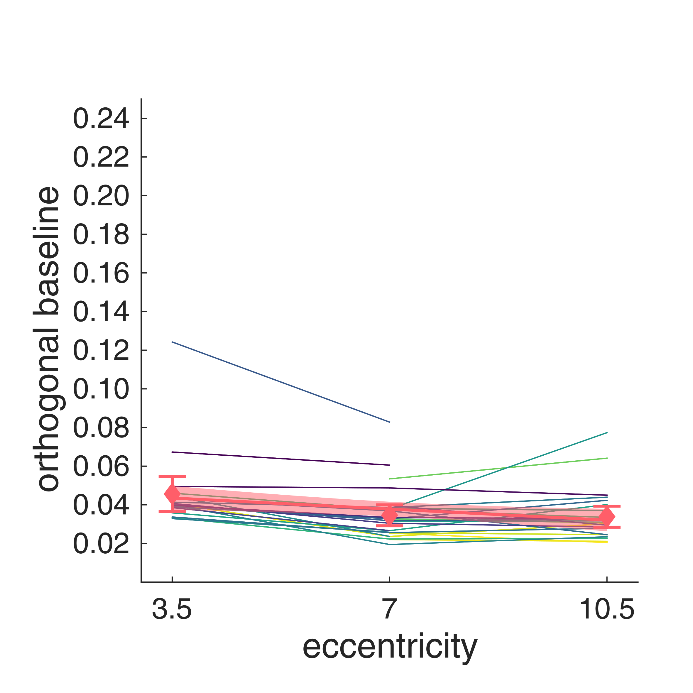

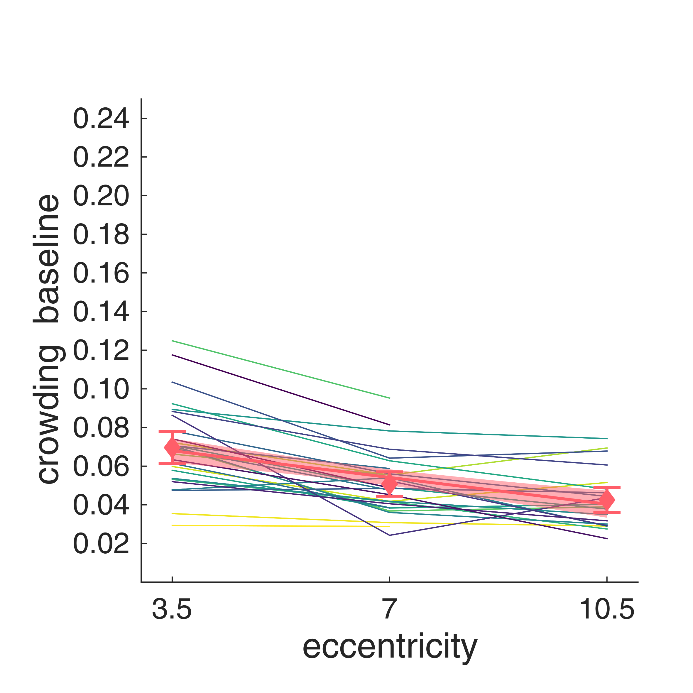

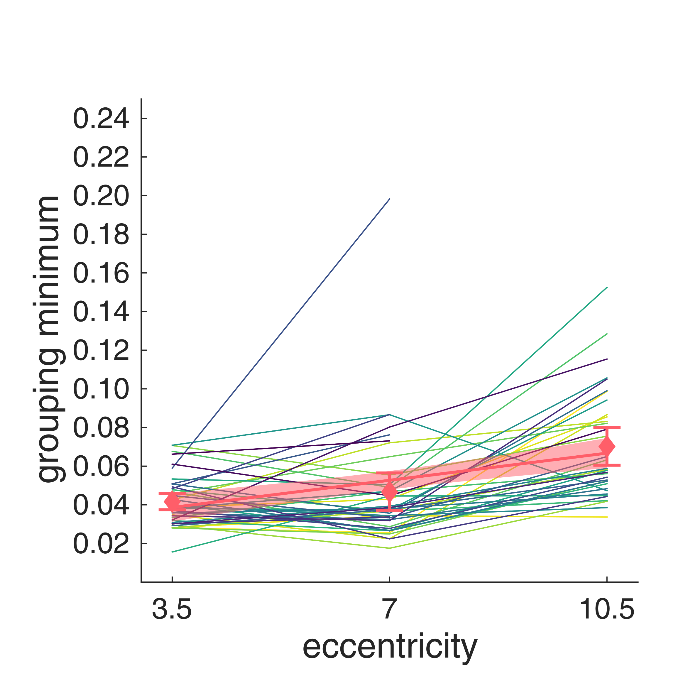


Supplementary Figure 1.2: Normalised baseline contrast as a function of eccentricity for each of the main tasks Individual participant data is plotted (each participant represented by line with a unique colour) alongside the mean per eccentricity (diamonds with error bars: 95% CI) and a general linear model (pink line with a shaded 95% CI). Note that the normalised data is plotted over half the scale of the raw data depicted in Supplementary Figure 1.
