# Supplementary material for: Masking, crowding, and grouping: Connecting low and mid-level vision
Source: J Vis. 2022 Feb 11;22(2):7. doi: 10.1167/jov.22.2.7 (PMC8842520; doi:10.1167/jov.22.2.7)
Supplement: Supplement 3 [file jovi-22-2-7_s003.docx]

# Supplementary material 3 – Comparison of Repeated Measures and Classical Correlations

To assess the relationships between baseline contrasts and critical distances within and between the tasks, we used repeated measures correlations.

Compared to the more widely used correlation measures (e.g. Pearson correlation) that make inferences on links between variables on the basis of between-participants correlations from averaged or aggregated data, repeated measures correlations allow conclusions to be drawn about relationships between variables for which multiple paired repeated measures exist in the within-subject data. For example, we measured baseline contrast and spatial windows at three different retinal locations. This is an important distinction, as it means that repeated measures correlations can be applied to the analysis of non-aggregated data from a repeated measures design without violating the assumption of independence of observation. Furthermore, in addition to a violation of independence of observations, a classical correlation over aggregated data also leads to an inflation of the degrees of freedom if no correction is applied. For further detail on why repeated measures correlations should be the preferred solution when evaluating links between variables in repeated measures designs, see Bakdash & Marusich, (2017).

As Greenwood et al. (2017) used classical correlations on aggregated data to interpret relationships between spatial localisation, crowding and saccadic eye movements, we provide the analysis over the aggregated data here, alongside the results of the repeated measures correlations that we reported in the manuscript (Supplementary Table 2.1). Repeated measures correlations on critical distance data do not show any robust correlations between the two flanker conditions of the masking task, or between them and the crowding task. Yet, when a classical correlation analysis is used to explore the relationships between the spatial extents of the different tasks moderate positive correlations are found that would be interpreted as indicating that participants that experience flanker interference from a farther distance in one task also do so in the other tasks.

**Supplementary table 2.1:** Comparison between the results of the repeated measures correlations and the correlations over aggregated data.

|  |  |  | Repeated measures correlation | | | Classical correlation over aggregated data | | |
| --- | --- | --- | --- | --- | --- | --- | --- | --- |
|  |  |  | df | r | p | df | r | p |
| Baseline | collinear | orthogonal | 33 | .709 | **<.0001** | 63 | .793 | **<.0001** |
|  | collinear | crowding | 37 | .412 | **.009** | 71 | .478 | **<.0001** |
|  | orthogonal | crowding | 28 | .507 | **.004** | 58 | .615 | **<.0001** |
|  | collinear | grouping | 52 | -.100 | .474 | 88 | .026 | .811 |
|  | orthogonal | grouping | 40 | -.279 | .073 | 73 | .120 | .307 |
|  | crowding | grouping | 45 | -.473 | **.001** | 81 | .080 | .474 |
| Critical distance | collinear | orthogonal | 34 | .291 | .086 | 66 | .407 | **.001** |
|  | collinear | crowding | 40 | .254 | .104 | 74 | .308 | **.007** |
|  | orthogonal | crowding | 28 | .392 | .032 | 57 | .377 | **.003** |
| collinear | Critical distance | baseline | 55 | -.354 | **.007** | 91 | -.240 | .021 |
| Orthogonal |  |  | 42 | -.318 | .036 | 75 | .075 | .519 |
| Crowding |  |  | 48 | -.023 | .874 | 83 | .277 | .010 |


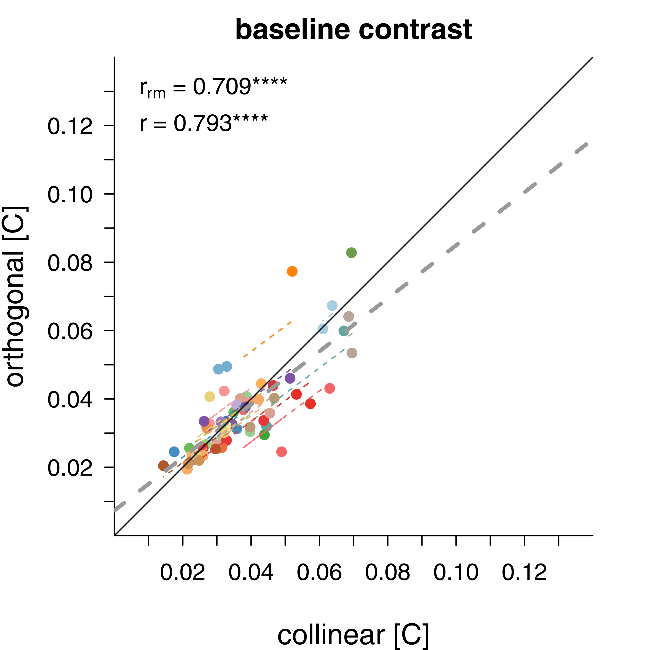

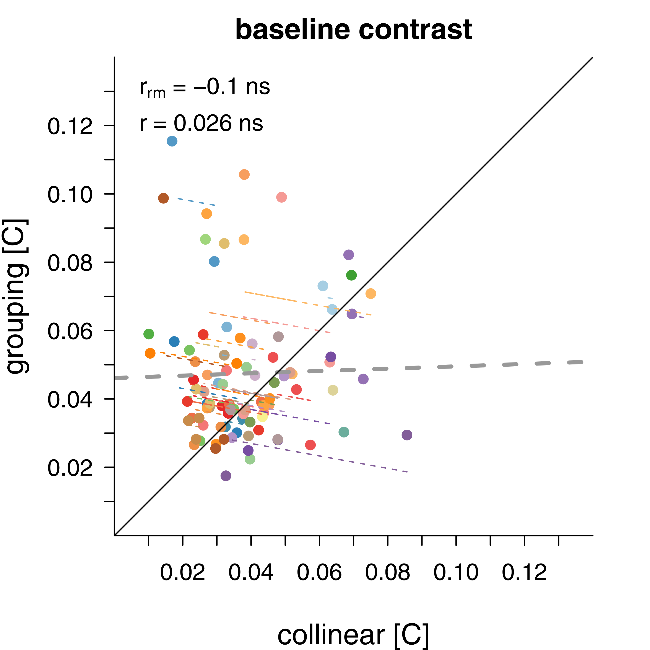

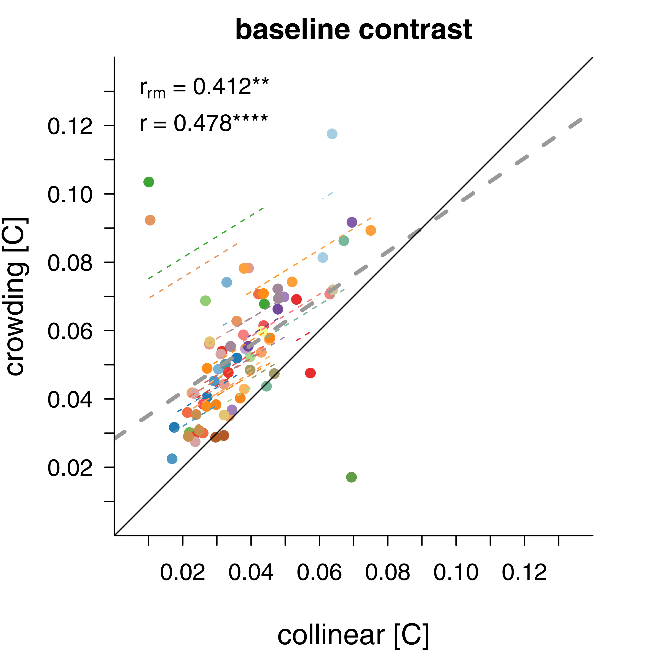

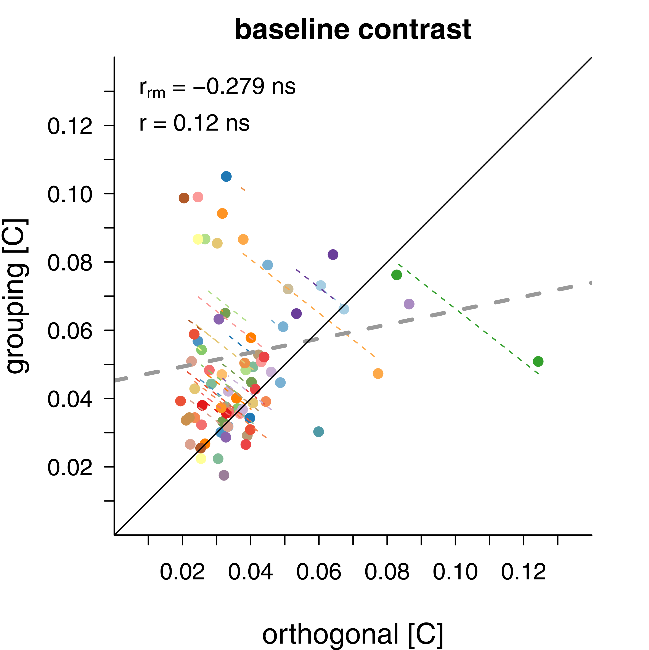

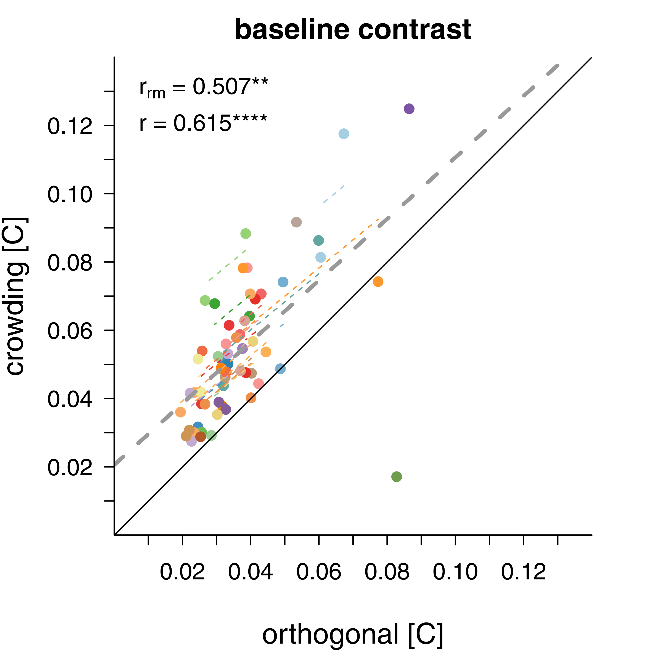

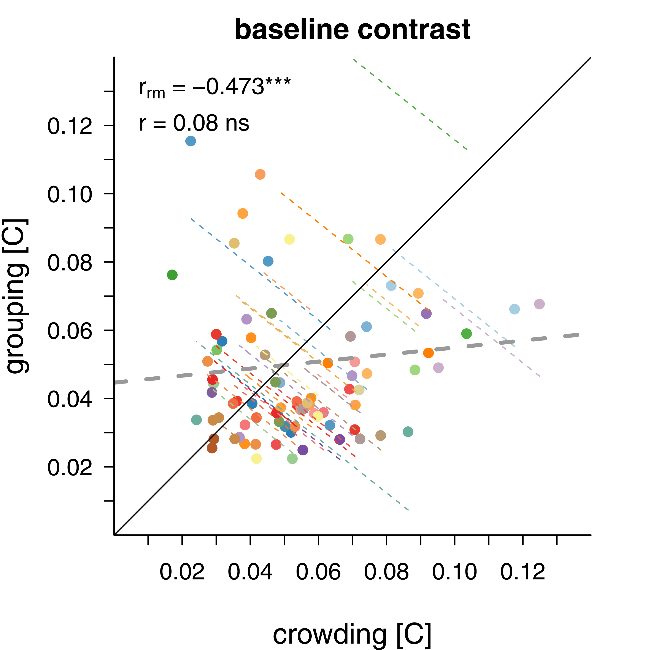


**Supplementary Figure 2.1:** **Correlations for baseline contrast.** Correlations are shown between all tasks. Dots indicate individual thresholds. Matching colours within a plot indicate that the thresholds stem from the same participant. Thin dashed lines show the correlations for individual participants over 2-3 threshold pairs for the repeated measures correlation. The thick dashed line shows the classical correlation on aggregated data, i.e. treating all data points as if they were individual measurements.


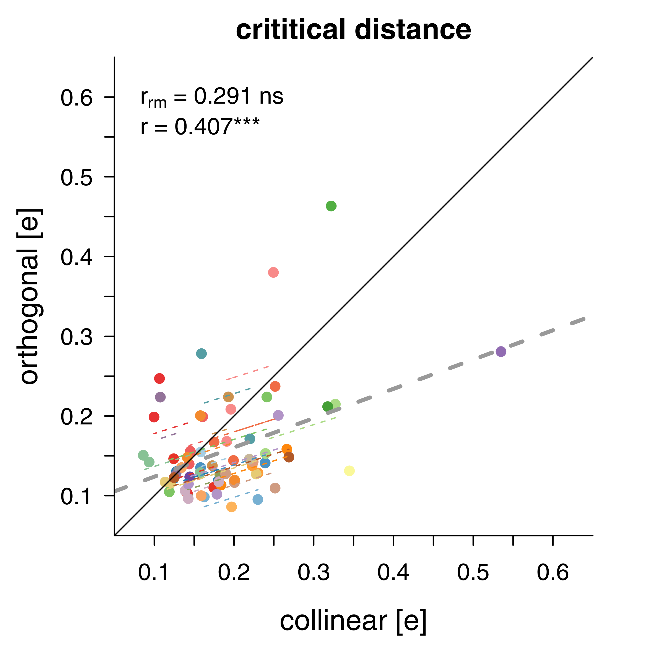


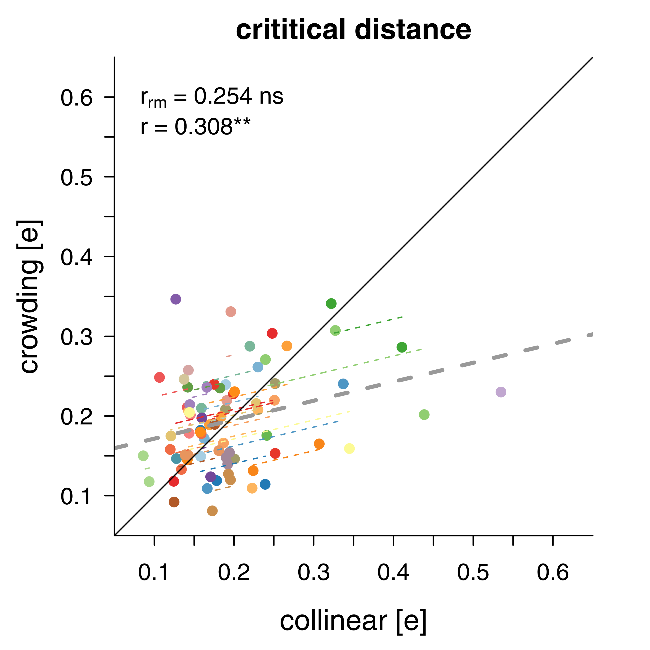

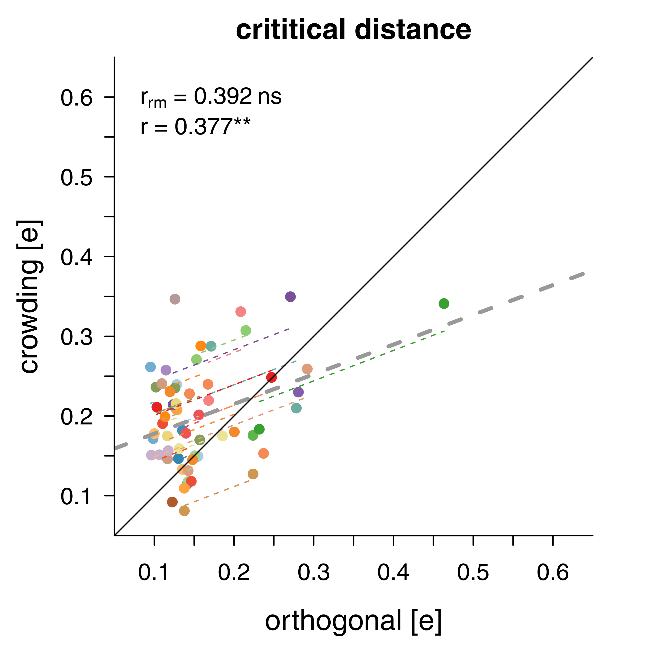


**Supplementary Figure 2.2:** **Correlations analyses for critical Distance.** Correlations are shown between both flanker conditions of the masking task and the crowding task. Dots indicate individual thresholds. Matching colours within a plot indicate that the thresholds stem from the same participant. Thin dashed lines show the correlations for individual participants over 2-3 threshold pairs for the repeated measures correlation. The thick dashed line shows the classical correlation on aggregated data, i.e. treating all data points as if they were individual measurements.


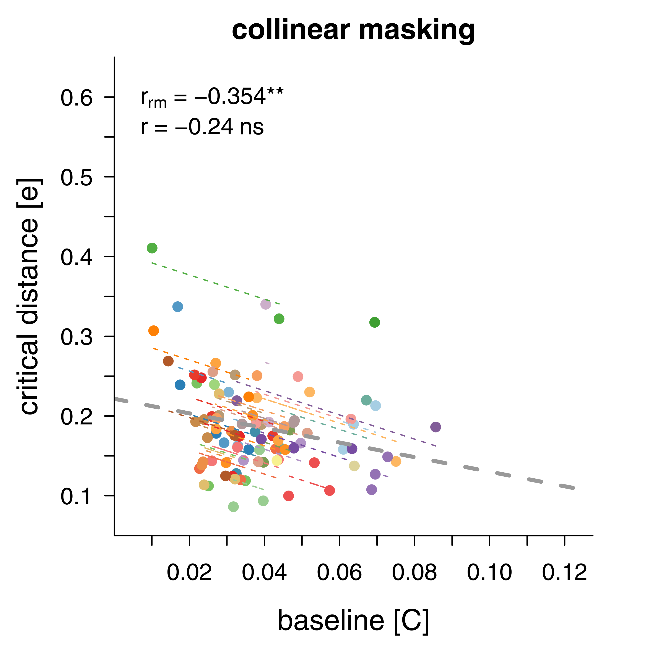

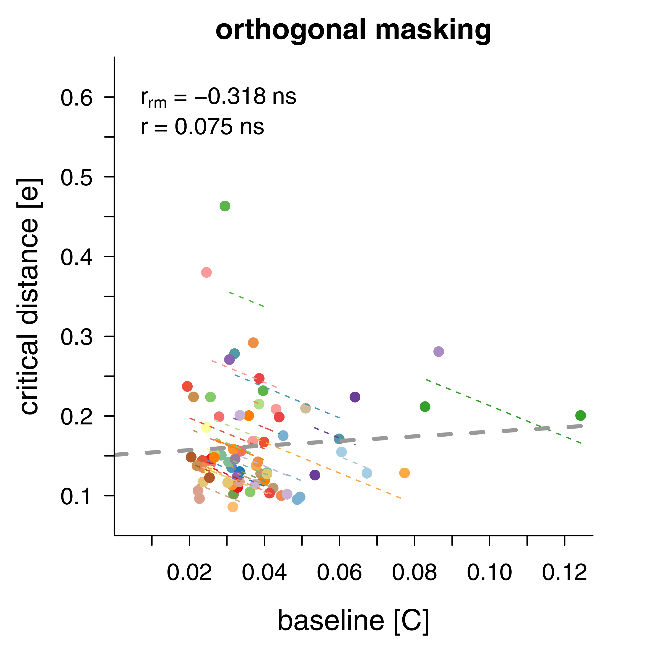

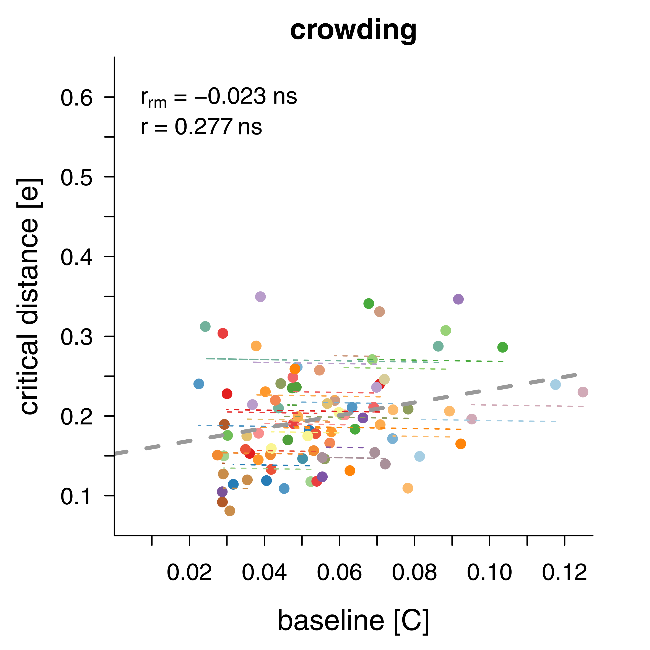


**Supplementary Figure 2.3:** **Correlations analyses for baseline contrast and critical Distance.** Correlations are for both flanker conditions of the masking task and the crowding task, respectively. Dots indicate individual thresholds. Matching colours within a plot indicate that the thresholds stem from the same participant. Thin dashed lines show the correlations for individual participants over 2-3 threshold pairs for the repeated measures correlation. The thick dashed line shows the classical correlation on aggregated data, i.e. treating all data points as if they were individual measurements.
